# Climate change, trending outcomes for the care of older people, and financial expenditure: a systematic review and narrative synthesis

**DOI:** 10.1186/s12889-026-27435-9

**Published:** 2026-04-23

**Authors:** Keriin Katsaros, Annika Traue, Jo-Ting Huang-Lachmann

**Affiliations:** 1 Climate Service Center Germany (GERICS), Helmholtz Zentrum Hereon, Hamburg, Germany; 2 Department of Global Health, Institute of Public Health and Nursing Research, University of Bremen, Bremen, Germany; 3 Institute for Chemistry and Biology of the Marine Environment, Carl Von Ossietzky Universität Oldenburg, Oldenburg, Germany

**Keywords:** Systematic review, Narrative synthesis, Climate change, Long-term care, Financial expenditure, Older people

## Abstract

A systematic review and narrative synthesis was completed to explore the relationships between climate change, the care of older people, and corresponding expenditure. While literature on these topics exists individually, a gap remains in linking all three. The interconnected challenges of an aging global population with increasing care needs and the rising frequency and severity of climate and weather events emphasize the urgency of minimizing related risks and their financial costs. Three databases were systematically searched from 1980–2023, identifying 36 peer-reviewed articles. Evidence suggests that climate and weather exposures lead to spending for recovery, preparedness, lifestyle, public administration, health and care, and temperature regulation.

The narrative synthesis, structured around the disaster management phases of preparedness, response, and recovery, revealed several behavioral, environmental, economic, social, sociodemographic, and structural determinants that appear to influence older peoples’ reactions to climate and weather events and financial consequences. Three key influences present across the narrative seem to shape spending and care outcomes: having strong support systems, access to user-friendly information, and existing economic resources.

Available literature on preparedness and recovery was limited, as most studies related to response expenditure. Future research should adopt a life-course approach to aging and preparedness using longitudinal designs to investigate how behaviors and choices made earlier in an individual’s life affect care, response, and recovery expenditure in older age. More stratified results would further allow for analysis of vulnerable subgroups and a clearer understanding of when climate and weather-related expenditures emerge. This review indicates the need for research and policy to consider the interconnected nature of the climate crisis. More focus should be given to preparedness efforts and behavior change to reduce the impact of climate and weather events on future expenditure and care needs.

Other

The protocol for this research has been published in PROSPERO with the registration number CRD42023443060.

**Supplementary Information:**

The online version contains supplementary material available at 10.1186/s12889-026-27435-9.

## Introduction

The impacts of climate change on the environment and human health are well-documented and acknowledged by key international organizations such as the United Nations (UN), World Health Organization (WHO), World Meteorological Organization (WMO), and the Intergovernmental Panel on Climate Change (IPCC), who have integrated these connected challenges into internationally accepted action plans, policy frameworks, and global objectives aimed at mitigating harmful outcomes [1–4]. WHO reports that climate change will be responsible for an additional 250,000 deaths per year worldwide from 2030–2050 [5]. This number, in combination with the current demographic transition to a globally older population, draws attention to growing concerns for existing challenges including the increased need for healthcare services, affordable and age-friendly housing, and social and caregiving support [6, 7].

Aging is typically associated with frailty and decreased physical and mental health and capabilities [7–9]. As the frequency and intensity of climate-related events increase, the challenges of providing appropriate care for older people with declining capabilities will be exacerbated [3]. Older adults in Africa and Asia, where there are limited response capacities to respond to climate challenges and rapidly growing aging populations are expected to experience even higher daily temperatures [10]. Seniors, particularly those living in low-income and lower-middle-income countries of the Global South, will face increased marginalization and inequality. This points to demographic change as an additional risk for aging societies as the climate crisis intensifies [11–13].

Independent of the impacts of climate change, the Organisation for Economic Co-operation and Development (OECD) indicates a steady rise in spending on health and long-term care in OECD countries driven by increasing costs of healthcare services and the demand of these aging populations [14]. They also report that without intervention, the economic effects of climate change on health due to extreme events, especially for vulnerable groups like older individuals, will continue to rise [15]. The 2023 Lancet Countdown on health and climate change emphasizes a detrimental increase in heat-related mortality since 2000 for people over 65 years of age coupled with an increase in economic burden for healthcare costs [16]. These projections are cause for serious concern: the World Economic Forum estimates the attributed losses to reach $12.5 trillion by 2050 [17].

There are existing systematic reviews and research on climate change, the care of older people, and expenditure, however there is a gap in literature linking the collective impact of all three challenges together. The main objective of this review is to explore the relationships between climate change, care of older people, and financial expenditure. Specifically, we examine how financial expenditure relates to climate change events and the care of older people, as well as existing trends and evidence at the intersection of climate, health, and care. These issues are deeply interconnected, with each having significant direct and indirect impacts on each other [18]. This systematic review provides insight into understanding broader policy and equity implications and how these interact with care systems, human health, vulnerability, and economic budgets as aging populations adapt to more extreme and frequent climate and weather events.

In this systematic review we synthesize findings from 36 peer-reviewed publications on climate and weather exposures, care, and expenditure among adults aged 50 and older. We identify existing gaps in literature and provide new insight into how we can explore the cumulative challenges addressed by leading global institutions. This work responds to the intersection of several Sustainable Development Goals (SDGs)^1^, economic expenditure and health impacts relating to the six weather-related disasters highlighted by the sixth IPCC report (2023) [4], and the Priority 1 of the Sendai Framework for Disaster Risk Reduction (understanding disaster risk) [19]. We present the results as a narrative synthesis structured by the disaster risk reduction phases, which provides a comprehensive overview of the included studies and identify gaps in the existing research.

## Methods

To ensure reproducibility, transparency, and rigor of this systematic review, the methods used are documented in the following sections. The review was conducted according to the guidelines and checklist in the 2020 Transparent Reporting of Systematic Reviews and Meta- Analysis (PRISMA) statement [20]. The completed PRISMA 2020 checklist for this review is found in supplementary document 1. Methodology was guided by recommendations from Cochrane and in the *WHO Guidance on research methods for health emergency and disaster risk management* [20, 21]. A study protocol was developed and published in PROSPERO with the registration number CRD42023443060 [22].

### Definitions and concepts

First, definitions for *climate change*, *older people*, and *care* were defined to inform the subsequent methodological components and ensure consistency throughout the review.

*Climate change*: we adopted the United Nations (UN) and the Intergovernmental Panel on Climate Change (IPCC) definitions, which refer to long-term shifts in temperatures and weather patterns due to natural variability or as a result of human activity [23, 24]. This review will consider all types of climate change, extreme weather events, and weather conditions.

*Older people*: To investigate trends in evidence on care and to represent older people in developing countries of the Global South with lower life expectancies, this review includes adults aged 50 and above [6, 25, 26].

*Care*: we adopted definitions of long-term care in relation to aging from the WHO and the National Institute on Aging (NIH), which explain long or short-term support with day-to-day personal, social, medical, and household activities either at home or in a facility provided either formally or informally by caregiver(s) [27, 28]. This review includes situations in which people “age in place”, or “the ability to live in one’s own home and community safely, independently, and comfortably, regardless of age, income, or ability level” [29]. We adopt a broad definition which includes formal and informal services used to meet support needs.

### Eligibility criteria

Pre-determined inclusion and exclusion criteria guided by the PICOS criteria (population(s), intervention(s), comparator(s), outcome(s), and setting(s)) were used to define the scope of included studies [30]. To minimize bias, two authors (KK,AT) pilot tested the criterion using a random selection of studies and the criteria were refined until minimum variation in interpretation and a high level of consistency in study selection was achieved between the two reviewers [31].

The final inclusion and exclusion criteria are presented in Table 1. Included studies focus on human adults aged 50 and older and must present stratified results relevant to this age group. Eligible publications must be written in English, peer-reviewed, and report original findings on expenditure related to the definitions of climate change and care in Sect. " Definitions and concepts". Studies published between 1 January 1980 to 28 June 2023 from all countries are included. The year 1980 was chosen as a starting point because the scoping search (Sect. " Information sources") indicated that studies relevant to the review topic began to appear during this period, likewise, reflecting a time most relevant to current contexts.

**Table 1:** Inclusion and exclusion criteria for publications based on PICOS criteria

PICOS Criteria	Inclusion Criteria	Exclusion Criteria
Population(s)	- about human adults - aged 50 years and older - results show trends about adults aged 50 or older - stratified results pertaining to adults aged 50 and older	- non-human (e.g., animals) - younger than 50 years old - studies relevant to the care of disabled people, infants, or care of children - results of study are not stratified by the relevant age groups
Intervention(s)	- consistent with the definitions of climate change, care, and older person established for this study	- study is not consistent with the definitions of climate change, care, and older person established for this study
Comparator(s)	- must be a peer-reviewed publication - must have research results - must be written in English	- is not peer reviewed - does not have research results (e.g., position and discussion papers, editorials, commentaries, protocols, and calls for action) - is not written in English
Outcome(s)	- study results include expenditure relating to climate change or care of people aged 50 and over	- results not relevant to expenditure relating to climate change or specifically to the health or care of people aged 50 and over
Setting(s),	- all countries	- Study is about the organizational or social climate of a setting, does not refer to meteorological conditions
time range	- published between 1 January 1980 and 28 June 2023	- published before 1 January 1980 or after 28 June 2023

### Information sources

First, a scoping literature search was conducted to assess existing publications and identify relevant search terms related to climate change, the care of older people, and expenditure to formulate an effective search strategy. PubMed was searched for biomedical and life science publications, the Cochrane Library for existing systematic reviews and meta-analyses, and PROSPERO for registered review protocols and ongoing systematic reviews to avoid duplication.

Following the initial scoping search, a keyword search was conducted by KK in PubMed, Scopus, and Web of Science (original search date: 28 June 2023). These electronic databases index peer-reviewed, literature consistent with the study’s inclusion criteria, across multiple disciplines. During revision, a search syntax error was identified for Web of Science. The search was rerun only for this database on 6 November 2025, identifying no additional eligible studies.

### Search strategy

The search strategy encompasses search terms in four categories (climate change, care, expenditure, and older people) linked using “OR” between terms of similar meaning and “AND” to separate each component. The search strategy, which was developed for PubMed and adapted for other databases can be found in Table 2. The full search strategies used for all databases can be found in supplementary document 2.

**Table 2:** Search strategy

Search term component	Search strategy
climate change	("climate chang*" OR "climate emergenc*" OR weather* OR "global warming") AND
care	("assisted living" OR nurs* OR facility* OR home* OR retire* OR care* OR long-term OR "aging in place") AND
expenditure	(cost* OR expen* OR financ* OR spend*) AND
older people	(elder* OR adult OR senior OR geriatric OR "aged care" OR "older adult*" OR "older people" OR pension*)

### Selection process

One author (KK) extracted publications and stored them in Zotero (Version 6.0.30). Duplicate records were removed. KK screened the title and abstract for compliance and relevance to the research questions and inclusion/criteria. Studies deemed irrelevant were excluded and remaining studies were extracted to an excel spreadsheet. A second screening took place using full-text publications. Studies were marked with “include”, “exclude”, or “discuss” if the study’s eligibility for inclusion was uncertain or required consultation with other reviewers to reach consensus. A second author (AT) independently screened 20% (n = 85) of the full-text publications [32]. Lists were merged and discrepancies were resolved by discussion between the two reviewers. Studies required consensus by two reviewers for inclusion. Publications marked with “discuss” were resolved with consultation from a third author (JTHL). Interrater agreement using Cohen’s kappa coefficient was calculated in Excel to assess the level of agreement between the reviewers. A near perfect score was obtained (κ = 0.85), reflecting high consistency in study selection between the two reviewers [33]. Both reviewers (KK, AT) independently verified the final list of eligible publications. No ethical approval was needed as only secondary data was used.

### Data collection

Data from the final list of publications was compiled by the main author by reading through full-text publications and extracted to a summary of findings spreadsheet in Excel. Information recorded included publication, title, year, author, study location, design, sample size, type exposure, related expenditure, and main conclusions. Multiple eligible findings within studies were recorded.

### Risk of bias and quality assessment methods

A risk of bias and quality assessment of the individual studies was carried out independently by two authors (KK,AT) according to Cochrane [34]. Due to many different study designs included, adapted Critical Appraisal Skills Program (CASP) checklists were selected corresponding to each design type; the Mixed Method Appraisal Tool (MMAT) was used for mixed-method approaches [35, 36]. As the aim of this paper is to compile an overview of existing evidence, it was decided not to assign a numerical rating or define a threshold for excluding studies based on the assessment. However, the appraisal of each study was used to inform the interpretation of results, with greater weight given to studies having a strong methodology and clear reporting of results, as suggested by PRISMA [37].

### Synthesis of results

Using information recorded in the summary of findings table, descriptive statistics and visuals summarizing characteristics of the included studies were generated using Excel and Sankey diagrams. A narrative synthesis is used to summarize the findings of individual studies included in the analysis. Given the diverse range of research designs and outcomes observed across the included studies, narrative synthesis emerged as the most suitable approach for interpreting and explaining the overall results [38]. A quantitative meta-analysis, therefore, was not appropriate.

Narrative synthesis as outlined by Popay et al. was followed to provide a comprehensive summary about existing research [38]. This includes developing an initial theory and preliminary synthesis, exploring relationships between studies, and then using the quality assessments to generalize the strength of evidence in the synthesized data. Through this iterative process, themes were systematically derived and refined. Disaster management cycle phases, *preparedness, response*, and *recovery* are incorporated into the narrative synthesis to provide a temporal perspective and identify gaps in research. In response to climate change, *preparedness* describes proactive measures taken before weather events that promote response capabilities and minimize impacts; *response* refers to actions taken during and immediately after that mitigate negative outcomes; *recovery* focuses on post-event, rebuilding. reconstructing, and restoration measures [19, 39].

## Results

### Studies included in the systematic review

**Fig. 1:**
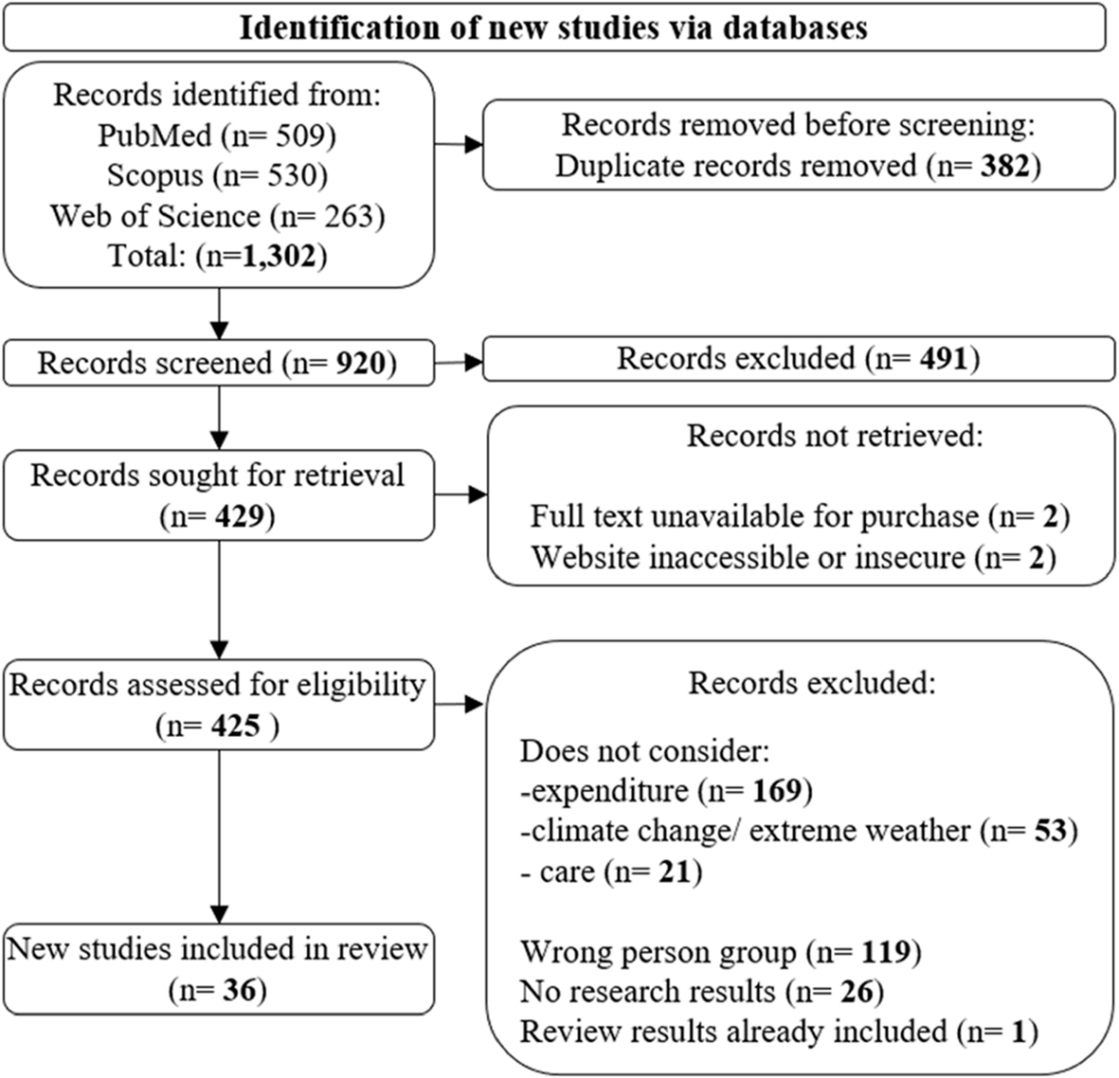
Flow diagram for systematic reviews according to PRISMA 2020

The PRISMA flow diagram depicting the study selection process is shown in Fig. 1 [37]. The final search identified 1,302 publications using PubMed (*n* = 509), Scopus (*n* = 530), and Web of Science (*n* = 263) between 1 January 1980 to 28 June 2023. 382 duplicate records were removed, leaving 920 publications to be screened. Title and abstracts of the remaining publications were screened for their relevance to the research questions and aims of the systematic review. 491 records were excluded and four could not be retrieved. A list of excluded studies and reasons for exclusion can be found in supplementary document 3. After excluding irrelevant papers 425 studies were screened, and 36 eligible papers were identified for inclusion in the review.

### Presentation of the selected studies

A summary of findings table presenting key information (location, study design, exposure, sample size, relevant age group(s), expenditure, and main outcomes) for each of the 36 publications included in the review can be found in supplementary document 4.

### Characteristics of included studies

This section summarizes and presents descriptive statistics for the location, publication year, the geographical scope of study samples, age distribution, climate exposures, and expenditure categories.

#### Location and publication year

**Fig. 2:**
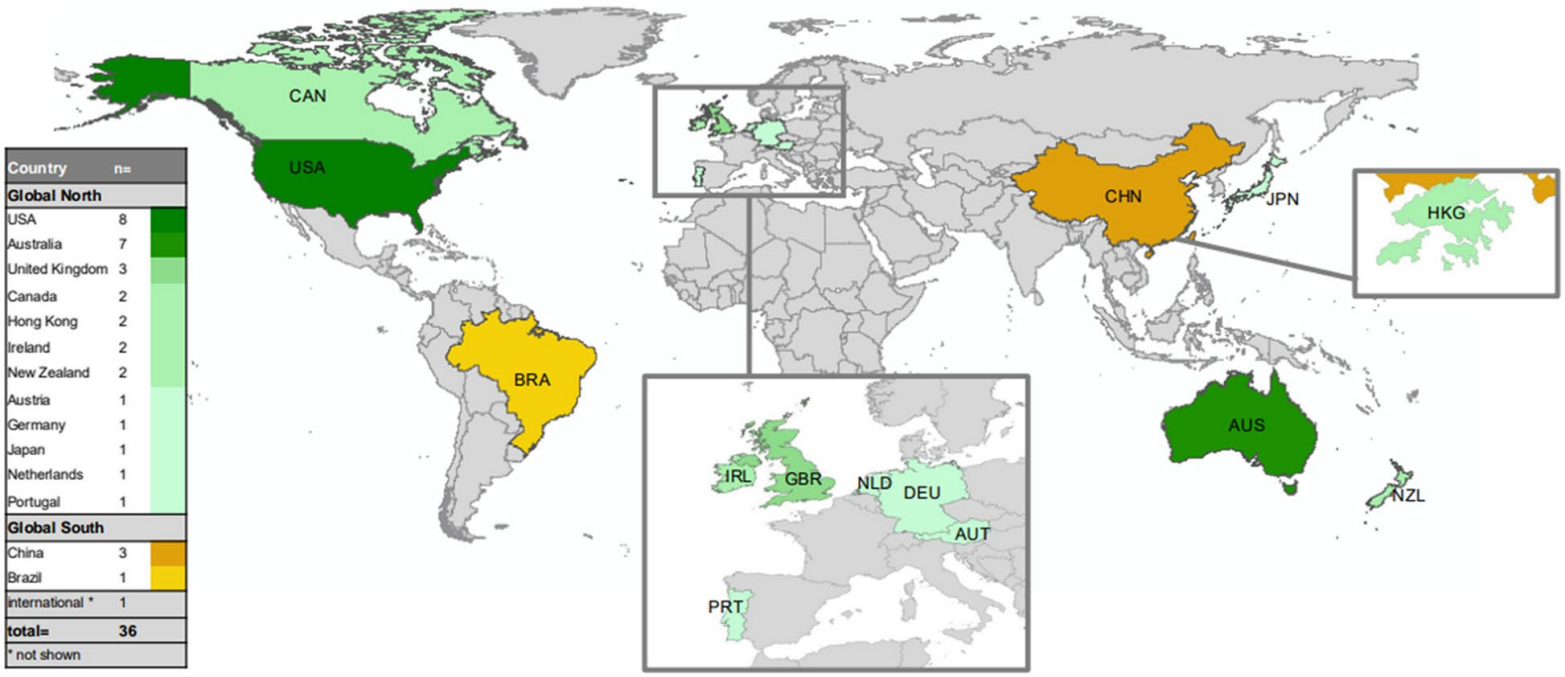
Location and number of studies included

Figure 2 shows the geographic distribution of the 36 publications. 31 originated from countries in the Global North, four were conducted in Global South, and one was an international study including countries from both. Fourteen total countries are represented in 35 individual publications. Country classifications were based on the World Bank country and lending groups. High-income economies are classified as Global North, while upper-middle, lower-middle, and low-income economies are designated as Global South [40].

Countries represented from the Global North include The United States of America (USA) (*n* = 8), Australia (*n* = 7), United Kingdom (*n* = 3), Canada (*n* = 2), Hong Kong (*n* = 2), Ireland (*n* = 2), New Zealand (*n* = 2), Austria (*n* = 1), Germany (*n* = 1), Japan (*n* = 1), Netherlands (*n* = 1), and Portugal (*n* = 1). In the Global South, China (*n* = 3) and Brazil (*n* = 1) are represented along with one international study that included multiple regions. Overall, the evidence collected in this review is concentrated from English-speaking countries in the Global North.

**Fig. 3:**
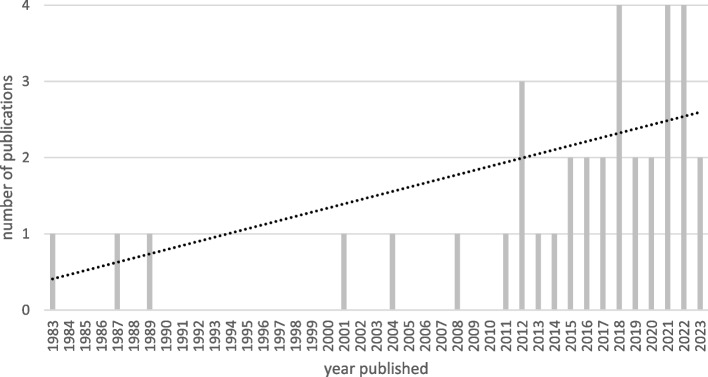
Number of publications by year for studies included in review

The publication years of included studies range from 1983 to 2023. Figure 3 shows the years of publication among the 36 included studies. Few publications appeared before 2010 (*n* = 6). Starting in 2011, there are publications included in each year, with an upwards trend represented by a dotted regression line showing an increased research interest in climate, care, and expenditure topics. The years representing the most publications in this review are 2018, 2021, and 2022, with four each per year.

#### Geographical scope of study populations

**Fig. 4:**
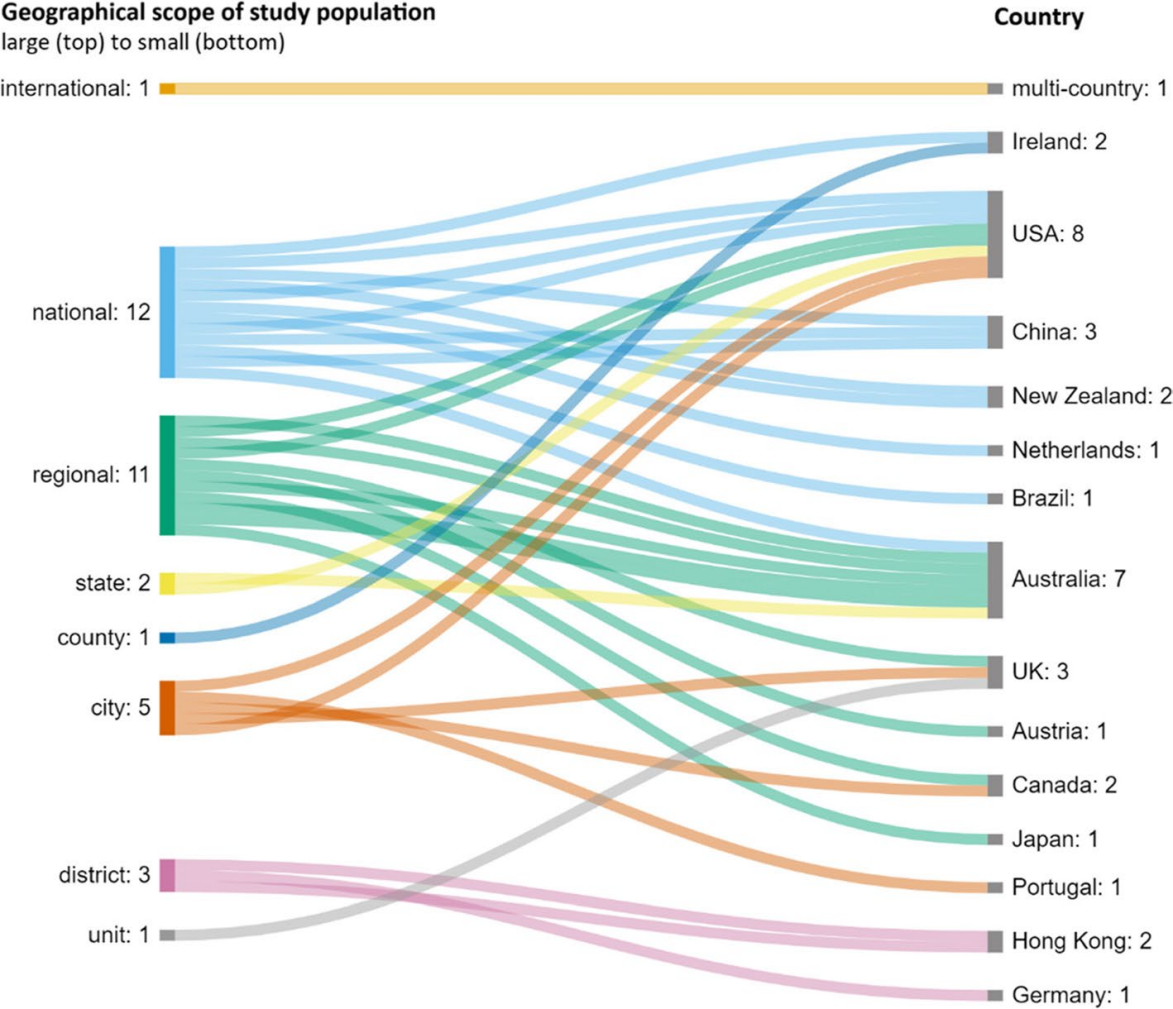
Geographical scope of study population in the included studies

The *geographical scope* refers to the scope of the study population that was recruited or analyzed in the study. National-level studies assessed data representing the whole country, while regional, state, county, city, district, and state levels examined smaller geographic areas within a country. Figure 4 illustrates the relationship between the study population’s geographical scope across the included studies and the country where it was conducted.

Most of the included publications examined study populations on the national (*n* = 12) and regional (*n* = 11) levels, followed by city (*n* = 5), district (*n* = 3), and state (*n* = 2) levels. Three studies focused on international (*n* = 1), county (*n* = 1), and unit levels (*n* = 1) (e.g., a single housing estate). The USA and Australia, which are most represented in this systematic review, include studies addressing populations on multiple scopes. Figure 4 shows that most of the studies included in this review represent large, national and regional populations.

#### Age distribution

The ages of older people in the 36 included studies were presented in several formats: as an average, range, hybrid interval (e.g. 50–65 +), or open-ended interval (e.g. 65 +). Two studies did not utilize numerical ages, rather referred to participants as “retirees” and “older people” [41, 42]. An overview of the ages presented across the studies is summarized in Table 3.

**Table 3:**
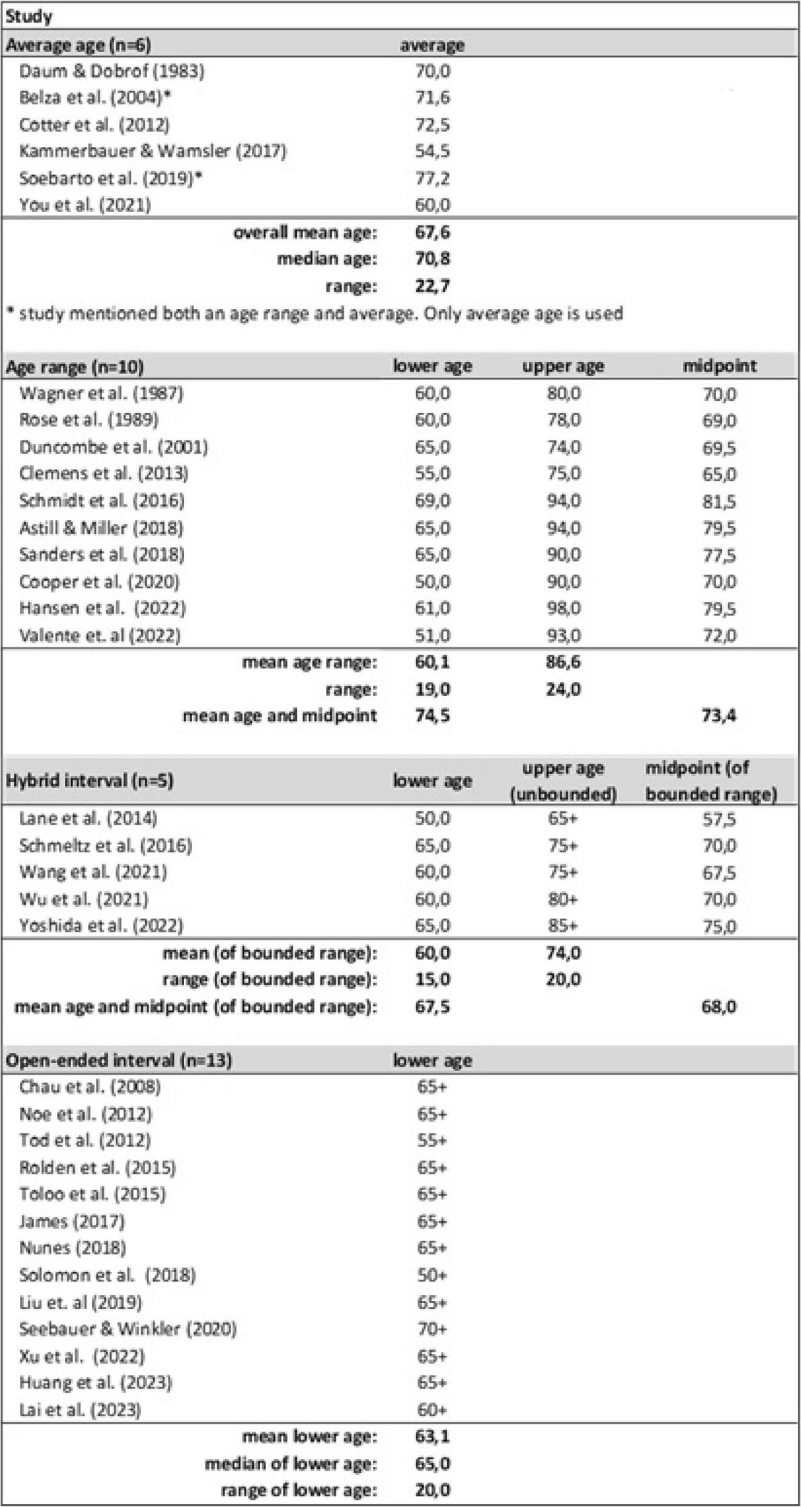
Overview of numerical ages across 36 included studies

Six studies reported the age of participants as an average. The mean average age was 67.6 years with a median of 70.8 years. Ten studies specified age ranges, with lower limits being between 50 and 69 and upper limits between 74 and 98 years old. Five studies reported age in a hybrid format with bounded lower limits of 50 to 65 and unbounded upper limits between 65 + and 85 +. Thirteen studies utilized only an open-ended interval that ranged from 50 + to 70 +. The study populations across the 36 studies represent older populations, yet the definitions and reporting of ages vary considerably.

#### Climate and weather exposures

This review included studies that addressed multiple climate- and weather- related exposures, with several studies addressing more than one event or condition. To enable the comparison of impacts and identification of patterns, the 16 different exposures identified across the 36 included studies were grouped into four broad exposure categories: *temperature*, *variability*, *other atmospheric phenomena*, and *precipitation*. Table 4 outlines the four categories and corresponding specific exposures identified, maintaining the terminology used in the original studies.

**Table 4:**
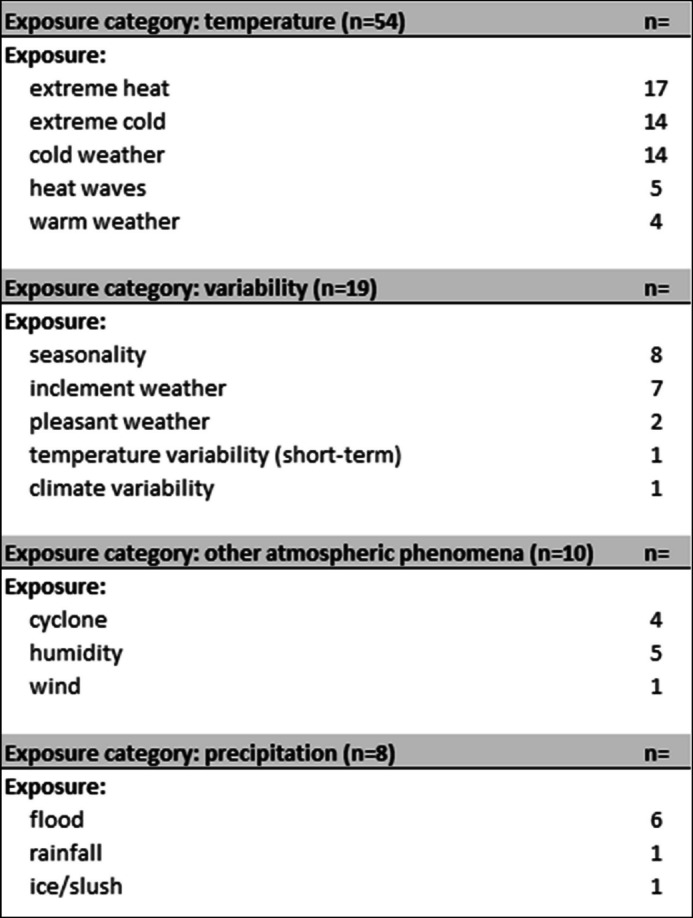
Climate and weather exposures represented in systematic review

The *temperature* category (*n* = 54) includes exposures relating to thermal conditions resulting in direct physiological or health effects. This category, representing the most common exposures in this review, includes extreme heat (*n* = 17), extreme cold (*n* = 14), cold weather (*n* = 14), heat waves (*n* = 5), and warm weather (*n* = 5). *Variability* (*n* = 19) is the second most represented category and includes seasonality (*n* = 8), inclement weather (*n* = 7), pleasant weather (*n* = 2), short-term temperature variability (*n* = 1), and climate variability (*n* = 1). These exposures describe fluctuations in climatic conditions rather than fixed temperature extremes, which affect behavior and health outcomes during non-extreme conditions. *Other atmospheric* phenomena (*n* = 10) describe climate and weather events not primarily related to thermal conditions or precipitation, such as cyclones (*n* = 4), humidity (*n* = 5), and wind (*n* = 1). Finally, *precipitation* (*n* = 8), encompasses exposures associated with hydrological events such as floods (*n* = 6), rainfall (*n* = 1), and ice or slush (*n* = 1).

**Fig. 5:**
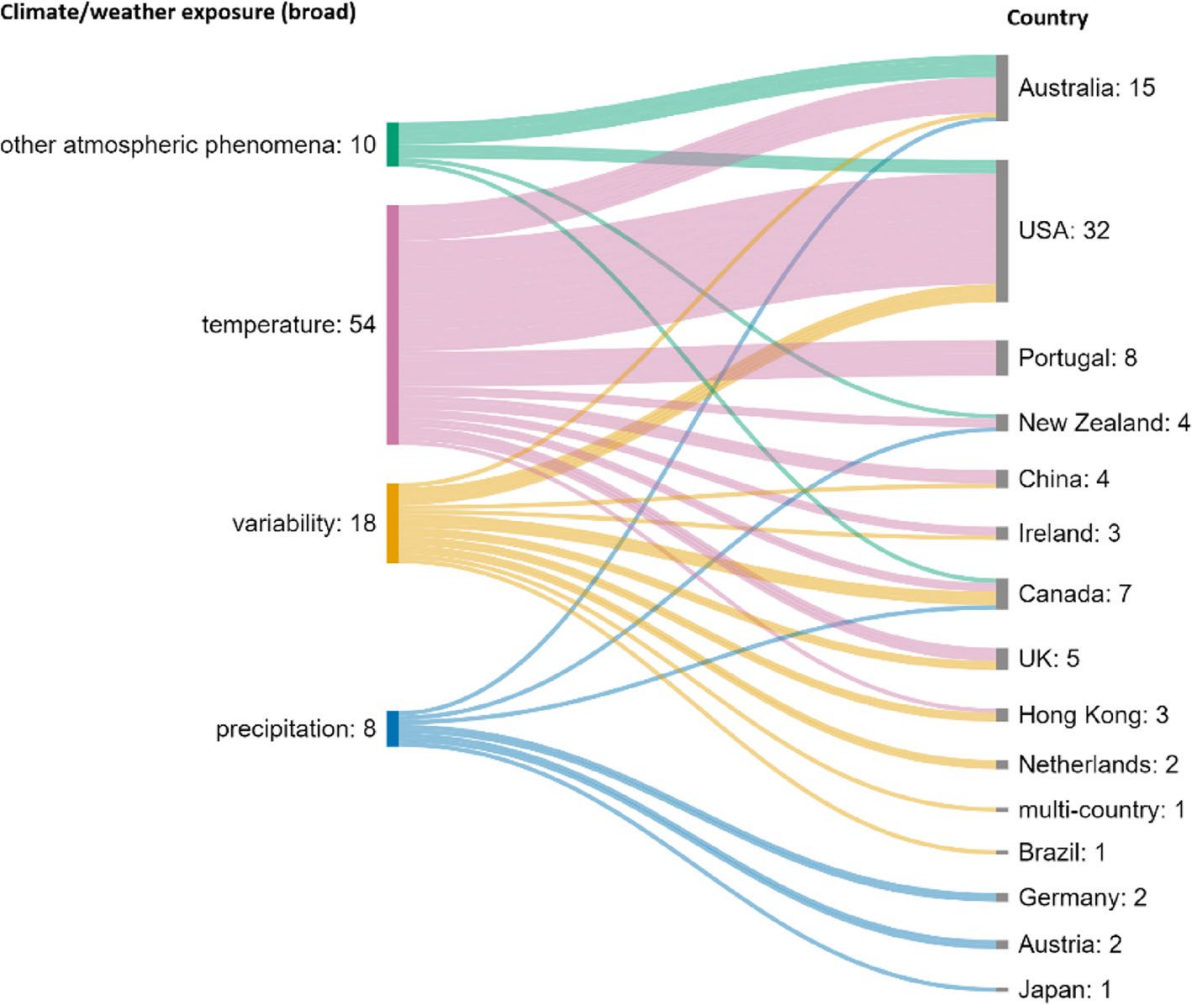
Exposure categories and country of research

**Fig. 6:**
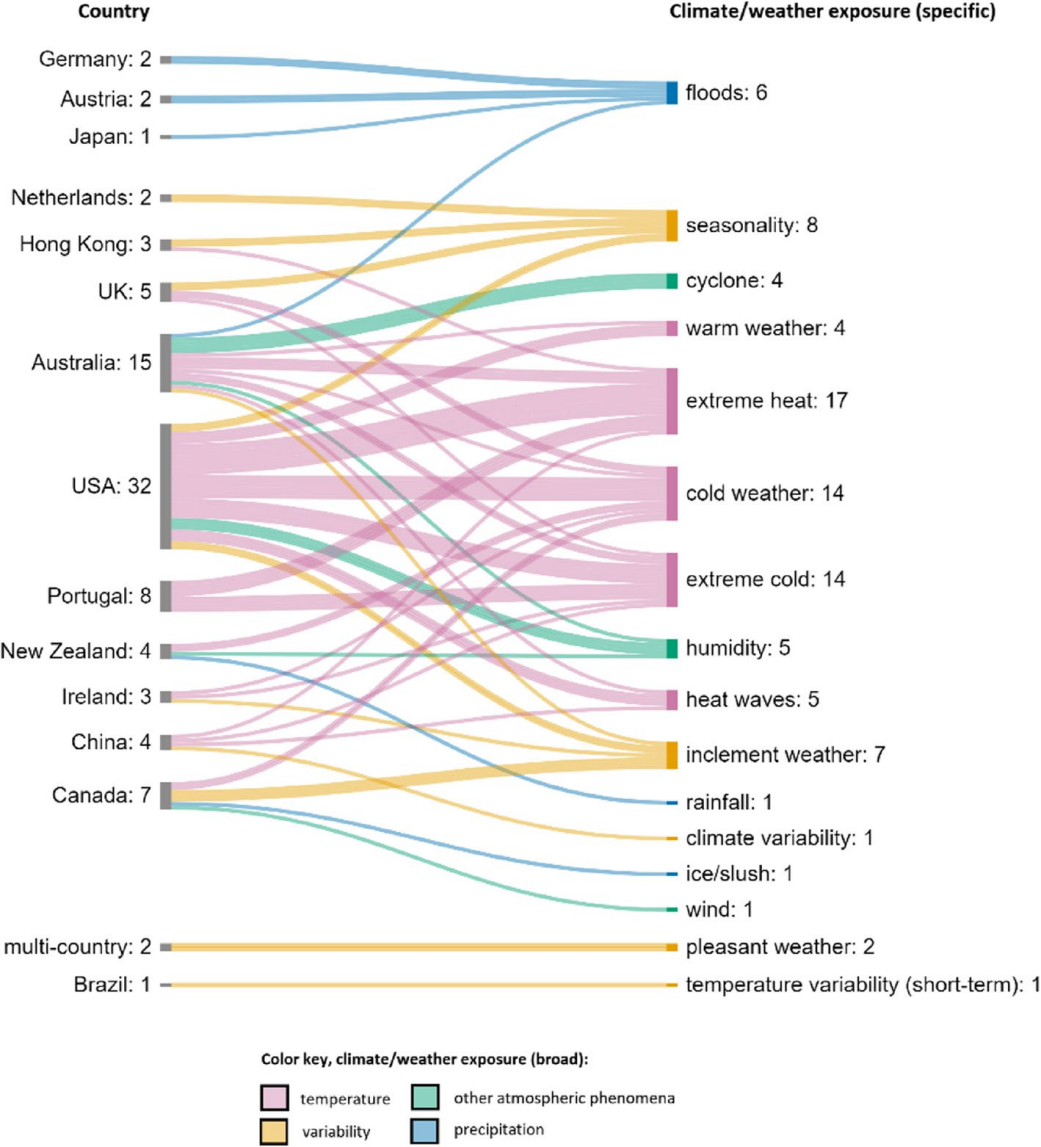
Country and specific exposure

Climate and weather exposures varied by country. Figure 5 shows how the broad exposure categories presented in Table 4 relate to the counties represented in the studies. The specific exposures represented in each country are shown is Fig. 6. Temperature was the most researched exposure by far (*n* = 54), followed by variability (*n* = 18), other atmospheric phenomena (*n* = 10), and precipitation (*n* = 8). Overall, most research represented focuses on temperature-related expenditure from countries in the Global North, specifically USA, Australia, and Portugal.

#### Expenditure related to climate and weather exposure

**Fig. 7:**
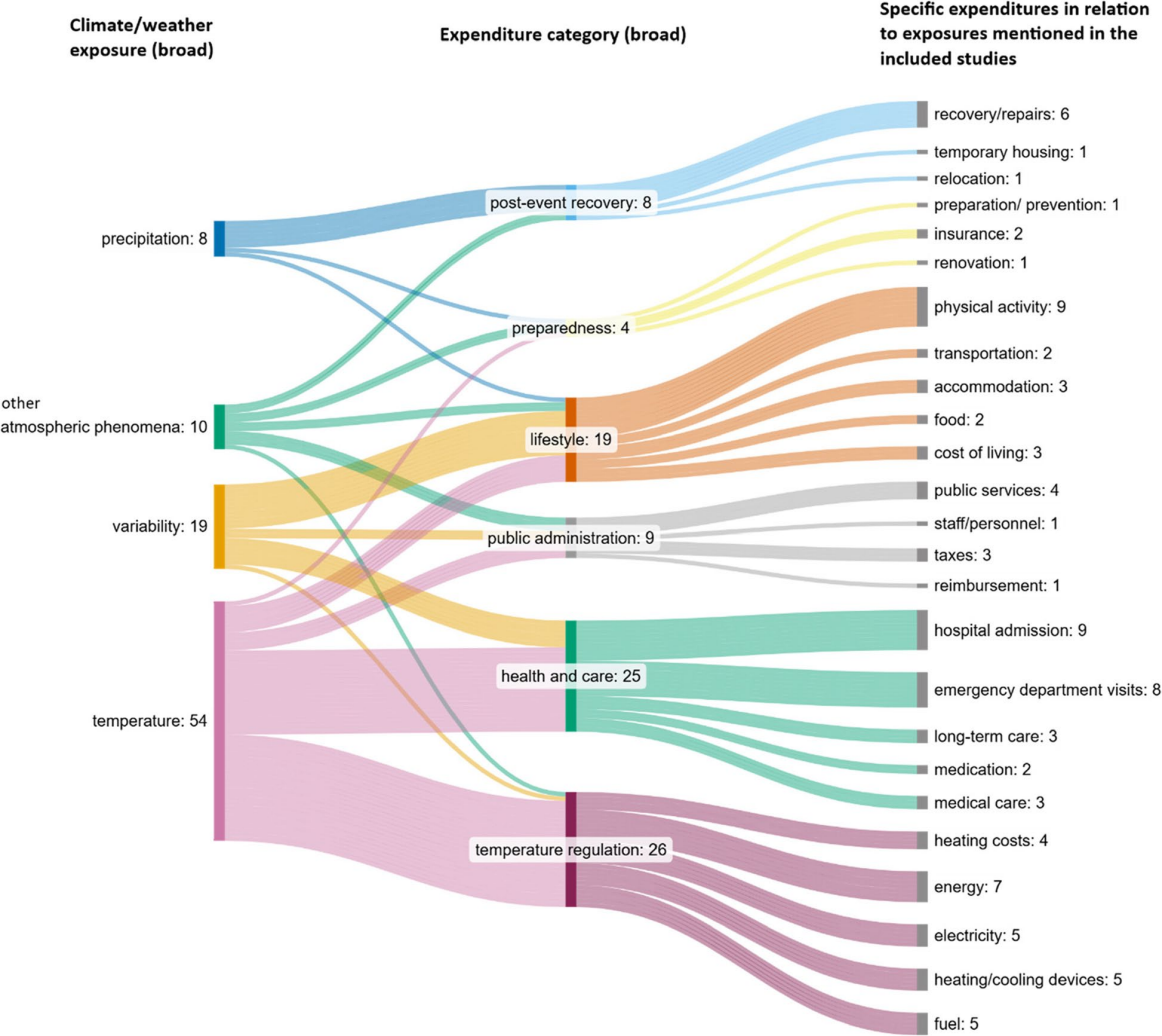
Expenditure resulting from climate/weather exposure

To better examine how climate and weather exposures relate to expenditure, specific expenditure types identified in the studies have been grouped into broad expenditure categories. Figure 7, below, visualizes the relationships between broad climate and weather exposure categories (Table 4) to expenditure. The terminology used to describe specific expenditures reflects the wording in the original studies.

In the included studies the largest exposure category, temperature (*n* = 54), was most linked to expenditure for temperature regulation, health, and care. Variability (*n* = 19) was primarily associated with lifestyle costs. Other atmospheric phenomena (*n* = 10) are associated with several types of expenditure including for post-event recovery, preparedness, lifestyle, and public administration. Finally, precipitation (*n* = 8) is associated primarily with costs relating to post-event recovery. Across all exposure categories, expenditure for temperature regulation (*n* = 26) and health and care (*n* = 25) were the most common followed by lifestyle (*n* = 18). Public administration (*n* = 9), post-event recovery (*n* = 8), and preparedness costs (*n* = 4) were least frequently reported.

**Fig. 8:**
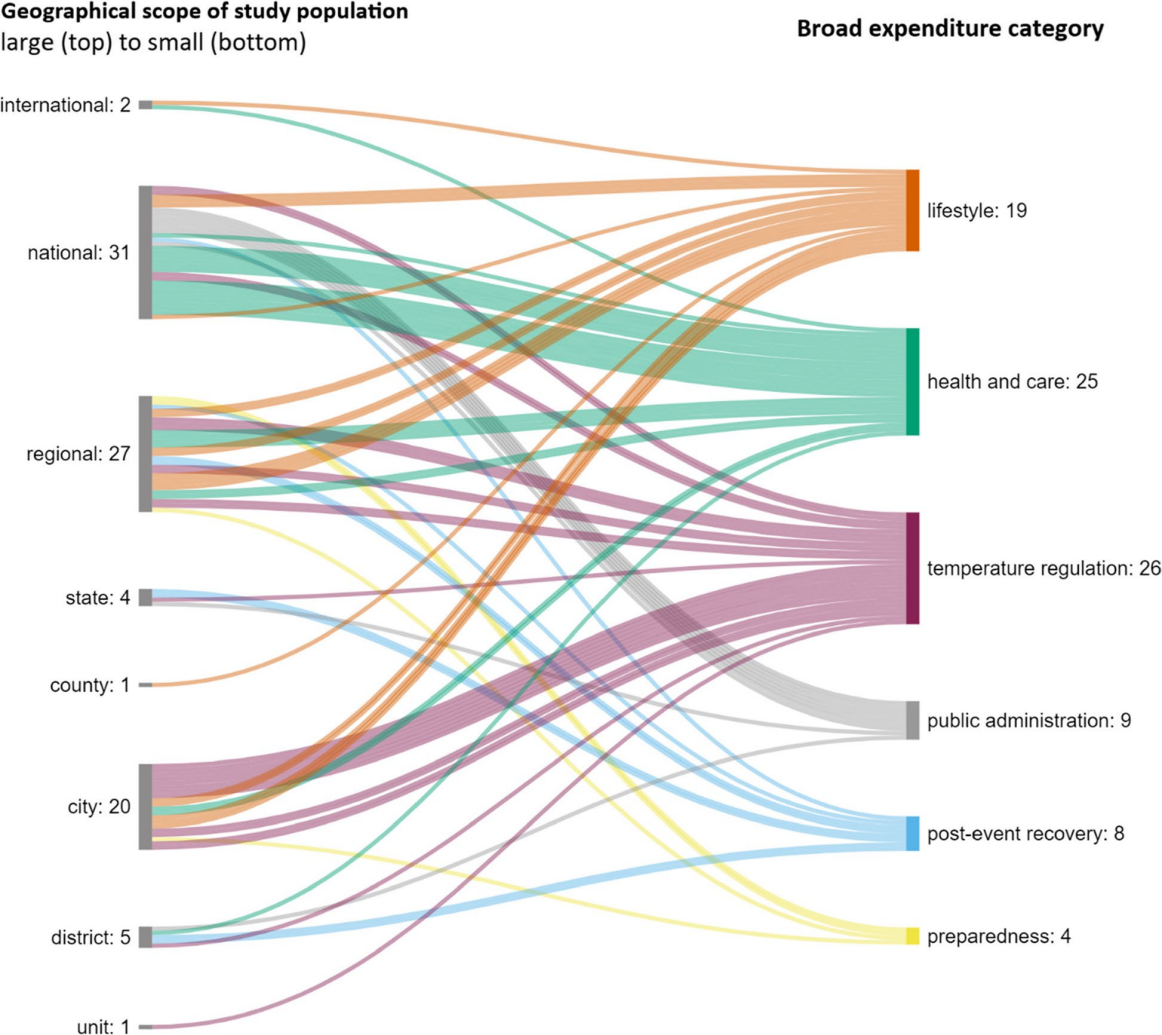
Geographical scope of study population and broad expenditure category

Figure 8 summarizes the relationship between the geographical scope of the study’s population and broad expenditure categories. National-level studies (*n* = 31) addressed expenditure primarily relating to health and care and public administration expenditure. On the regional level (*n* = 27), expenditure was distributed across all broad expenditure categories except public administration, with no dominant expenditure type. Expenditure for temperature regulation dominated city-level studies (*n* = 20). No studies relating to international, national, state, county, district, or unit levels addressed preparedness (*n* = 4). Similarly, public administration expenditure (*n* = 9) was not addressed on the international, regional, county, city, and unit levels.

### Results of risk of bias and quality assessment

CASP checklists for case control (*n* = 6), cohort (*n* = 9), economic (*n* = 1), qualitative (*n* = 10), systematic studies (*n* = 1), and the MMAT (*n* = 9) checklist were used to appraise the methodology and outcomes of included studies. The quality assessments are found in supplementary document 5.

### Narrative synthesis

This section presents a summary of findings emerging from a narrative synthesis that was conducted to assess the collective impact of challenges, examine relationships, explore trends and evidence, and identify gaps across the 36 included studies in this review. It maps out key patterns and relationships related to how climate change affects expenditure and care.

#### Framework

**Fig. 9:**
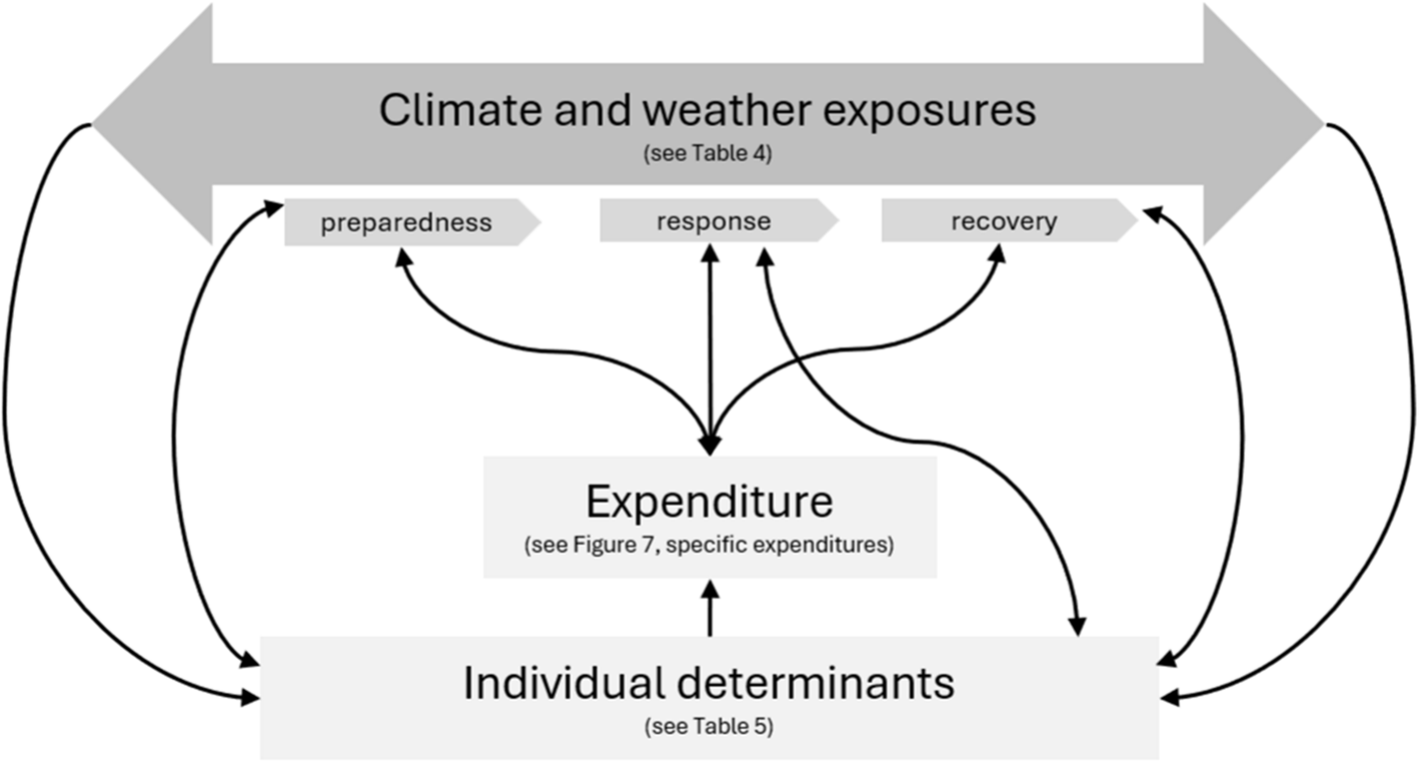
Framework of narrative synthesis

We found that climate and weather exposures, specifically the preparedness, response, and recovery from them influence both expenditure and individual determinants (Fig. 9). For this review, *individual determinants* are grouped into six overarching categories: behavioral, environmental, economic, social, sociodemographic, and structural. Individual determinants describe factors that shape how people respond and make decisions. *Attributes* within each individual determinant category are personal and contextual factors that affect expenditure, reflecting how older people and the systems they belong to prepare for, respond to, and recover from climate and weather exposures (Table 5). This narrative synthesis framework (depicted in Fig. 9) forms the structure of the subsequent sections and organizes the identified themes across the disaster risk reduction phases, following older adults as they prepare for, respond to, and recover from climate and weather exposures.

**Table 5:** Individual determinants affecting expenditure and response to climate and weather exposures in the included studies

**Individual Determinant Category**​	**Attributes**​
Behavioral​	• adaptive responses [41, 43–54] • beliefs [43, 44, 55] • emotions [56–59] • frugality [43–52] • perception of risk/coping​ [43, 44, 54, 57, 58] • physical activity level [42, 44, 54, 55, 60–64] • preference [42–44, 48, 54, 60–62, 65] • self-efficacy [41–44, 46, 48, 50, 51, 54, 57–59, 61, 66]
Environmental	• geographic location [42, 47, 57, 59, 65, 67]​ • housing [43, 46, 48–51, 54, 57, 59, 66, 67] • public infrastructure [58, 63] • public spaces​ [43–45, 49, 52, 54, 55, 61, 63]
Economic	• employment status [62] • home ownership [54, 56, 57, 66] • income/savings [42–46, 48, 49, 51, 52, 54, 55, 57, 58, 60, 61, 63–66] • insurance [58, 59, 66]
Social	• access to information​ [41–46, 48, 52, 54, 58, 59, 61, 63, 65, 66] • community [42, 55, 58, 59, 61, 63, 64] • family/friends [42, 45, 46, 52, 55, 58, 61, 63, 64] • social cohesion [59, 68]
Sociodemographic	• advanced age [45, 48, 63, 67] • culture [42, 55, 61, 63, 64] • health status​ [41, 42, 44, 45, 48, 49, 51, 54, 55, 60–64, 69] • language spoken​ [42, 63] • race [68, 70] • sex [70–72]
Structural	• energy supply​ [57, 59] • public programs​ [42, 45, 65] • social insurances [59, 67] • transportation [52, 55, 61, 63, 64]

#### Preparedness

In preparing for the impact of climate and weather exposures, the included studies examined the environments in which older people live. Several attributes influenced expenditure on the built environment and their decisions to either remain in place or relocate.

##### Alterations to the built environment

In the context of preparedness, included studies assessing the built environment focused on expenditure related to modifying existing buildings and building new structures. Our synthesis found that older people residing in dated buildings often have no central heating or air conditioning, poor insulation, and were constructed with features that are not conducive to cost-effective temperature management such as single-glazed windows and a lack of ceiling fans [48, 54]. Low quality, cheaper building materials used in some old homes in New Zealand were linked to leaky building syndrome. This term describes houses that are not weather-tight to precipitation [66].

Preparation for climate and weather impacts involved expenditure for modifications to existing buildings by weatherizing homes, retrofitting rooms against cold, and installing features such as solar panels, air conditioners, external blinds, ceiling fans, double-glazed windows, and insulated doors [46, 50, 51, 53, 66]. These improvements resulted in increased thermal comfort, temperature regulation, and reduced energy expenses. However, high renovation costs and landlord’s reluctance to invest in rented apartments were barriers. Two studies addressed considerations for new construction, including the location, placement, shape, and design of buildings to manage heat and the ability to construct a new, age-friendly home after relocating from a high-risk flood area [43, 57].

##### To stay or leave?

Older people are confronted with the decision to stay or leave their established residence prior to extreme weather events and in retirement. In two studies, the decision to remain in place was influenced by limited income or savings, an emotional attachment to their homes, rising housing costs, perception of their ability to cope, and the absence of a reliable support systems [57, 58]. According to one study, older people experienced financial strain when preparing for cyclones while staying in place. This was linked to the costs of purchasing extra food and flood insurance. Limited support from social networks, friends, and family, along with difficulties in accessing online information, further hindered their ability to prepare and recover [58].

Two studies on retirement migration found that older peoples’ relocation decisions were influenced by limited financial resources, preference for favorable weather conditions, more affordable living and healthcare costs, and access to better public infrastructure [42, 65]. They sought retirement locations with established expatriate communities that could serve as social networks to assist in navigating foreign services [42]. A separate study from Hong-Kong found that air quality affected by outdoor weather conditions prompted some older people to relocate to other districts. In these areas, they could avoid the expense of air purifiers by adopting behavioral measures like opening windows [47].

#### Response to climate and weather exposures

This section presents findings on how older people respond to climate and weather impacts and the associated expenditure with these responses. This phase accounted for most of the included evidence. It examines the implications for physical and mental health and for participation in physical activities. Furthermore, we outline how older people adopt cost saving strategies and how their cognition, preferences, and perception of their resilience shape these responses.

##### The impact of climate and weather exposures on physical health

The included studies report several adverse outcomes for older people caused by elevated environmental temperatures. These include tiredness, shortness of breath, sleeplessness, dizziness, overheating, and high blood pressure [43, 45, 51]. Lower environmental temperatures were likewise associated with arthritis and painful joints, increases in falls, colds, flu, respiratory illness, shortness of breath, decreased energy, and depression [49, 51, 62]. Fear of falling during winter and reduced physical activity were commonly mentioned [49, 54, 55, 62, 64]. Several studies illustrate the relationship between non-ambient and extreme temperatures and increased health care expenditure (e.g. for emergency department visits, outpatient care, hospitalization) [68, 70–75]. Similarly, variability and other atmospheric phenomena are associated with both respiratory ailments and increases in medical care costs [66, 69, 72, 76].

Studies mentioning multimorbidity and chronic conditions were linked to worsening medical conditions and overall health status. For example, high temperatures in combination with preexisting medical conditions were associated with greater susceptibility to illness, tiredness, and overheating [44, 45]. Low temperatures were associated with increased arthritis pain [49]. Fatigue, insomnia, and pain were reported by older women living with HIV experiencing intermittent weather- related ailments [62]. Older adults explained that their advanced age affected their circulation, making it harder to regulate temperatures [45, 48]. Older people from minority racial groups [68, 70] as well as men [70–72] were more likely to be hospitalized. Additionally, some chronic conditions left older people reliant on electricity- dependent devices, such as nebulizers, and oxygen and CPAP (continuous positive airway pressure) machines, which increased their electricity bills [41, 45]. While these studies demonstrated direct links between climate and weather exposures and physical health, a parallel narrative emerged revealing various ways older people adapted their behaviors to save money.

##### Behavioral adaptations for cost reduction

Older individuals engaged in several cost saving, avoidance, and trade-off strategies to limit their expenditure in response to climate and weather exposures. Various adaptive responses were used to save on cooling including opening windows, using fans, wearing lighter clothing, drinking more water and colder beverages, opening windows, closing blinds, putting cold towels on the body, and adjusting sleep schedules [43–47]. To stay warm, older people employed a range of cost-saving measures to reduce heating expenses. These included using blankets, rugs, and curtains, keeping doors closed, dressing in additional layers, reducing shower frequency, consuming warm beverages, and using space heaters and heat lamps [46, 48–52]. Two studies also reported that older individuals sought assistance from food banks [45] or went hungry to save money and be able to pay for fuel in the winter [48, 49].

In the same way, avoidance behaviors were used to control spending on heating and cooling, with some older people choosing to spend time away from their homes in more comfortable environments. Visiting air conditioned public places such as supermarkets, coffee shops, community centers, cooling centers, and shaded public places was practiced [43–45, 54]. In low temperatures, older adults frequented heated public places and senior centers [49, 52]. To afford electricity, some older people in Australia resorted to potentially harmful trade-offs, including forgoing medical appointments not covered by public insurance (e.g. dental, physiotherapy). Others abstained from purchasing hygiene products, healthy food, and some restricted their use of energy-dependent medical devices [45]. Older people on pre-paid electricity meters rationed their usage by turning them off during the day [41]. Sacrificing thermal comfort [53] or regulating temperature only in certain rooms to save on costs were additional trade-off adaptations [46, 48, 49, 53, 54]. Older people in these included studies may have managed to save money, but they did so at the expense of their health, comfort, and by organizing routines around maintaining thermal comfort.

##### Mental health impacts and financial strain

Across the included studies, a narrative developed linking older peoples’ savings and income to challenges in adapting to climate and weather exposures, negatively impacting mental health. For example, older adults faced challenges in purchasing climate appropriate clothing, medications, heating and cooling devices, and nutritious food [43–45, 48, 49, 52, 54]. Some mentioned having to unexpectedly use retirement savings to fix their homes, straining their available resources [66]. These response expenses were shown to cause high levels of worry among older adults with strained incomes, limiting their participation in social activities and contributing to increased feelings of isolation and loneliness [45, 46, 48, 49, 51, 56]. In one study, a lack of available transportation options in winter further hindered socialization as well as attending medical appointments [52]. Increased temperatures resulted in heightened irritability for some individuals. This contributed to less contact with family and friends, and declining mental health that further complicated the ability to mitigate heat [43, 48, 54].

##### Individual preference and risk perception

In several studies, personal preference and perceived resilience motivated the adoption of cost-saving behaviors. Three studies reported that older people either disliked the sensation of air conditioning or preferred using fans instead [43, 44, 54]. Some beliefs were influenced by the perspective of Chinese medicine, which suggests that prolonged exposure to air conditioning leads to fatigue and an increased risk of developing arthritis [43, 44]. Thriftiness was emphasized by others who viewed air conditioning as economically wasteful. These older people took pride in their frugality, which enabled them to maintain independent living [43, 45, 48]. Personal risk perception was a strong factor in engaging in temperature-regulation behaviors. Some older people did not perceive themselves as vulnerable to heat or did not view it as a threat to their health [43, 44, 54]. Others, rather, believe that lower temperatures pose a threat [43].

##### Participating in physical activities

Engagement in physical activity arose as a distinct behavioral attribute mentioned across several of the included studies. Physical activity was identified as important for maintaining mobility and fostering social connections amongst older people, with participation influenced by weather conditions, program costs, transportation, and perceived benefit. Outdoor exercises like walking and biking were preferred by many [42, 60–62]. However, inclement weather conditions (e.g. low temperatures, rain, and cloudy weather) and purchasing appropriate clothing acted as barriers to outdoor physical activity [55, 60, 63]. Likewise, available public infrastructure and the physical environment were linked to influencing older adults’ participation. One study found that walkable outdoor spaces, natural environments, and the availability of fitness centers in the community acted as enablers while barriers included a lack of parks and crowded streets [63]. The safety of public spaces was a common concern mentioned by older people across many studies [44, 54, 55, 61, 63].

Perceived benefit and costs of gym memberships, exercise classes, and reliable transportation were shown to strain retirement budgets and limited participation in indoor physical activities during unfavorable weather conditions [55, 61, 63, 64]. Although age and underlying health conditions were described to limit and motivate participation in physical activities, older people connected being active with improved mental health and cognition [55, 60, 61, 63, 64]. In several reviewed studies, individuals were motivated to exercise and adhere to physical activity programs when they were given the opportunity to engage with members of the same cultural and religious groups. This provided peer support, reduced language barriers to obtaining information, and fostered a sense of community belonging [55, 61, 63, 64]. Engagement from family was found to be essential in promoting physical activity and supporting the maintenance of an independent lifestyle [55, 61, 63, 64].

##### Cognition

In response to climate and weather events, cognition reflects how older people obtained, processed, and used new information in their decision-making responses. Information was drawn from a variety of channels. Older people tended to trust details on fuel pricing and physical activities when they came from familiar contacts and sources. Regarding temperature and how to prepare accordingly, information was received from public radio, community officers, television, and medical professionals [43, 44, 54]. Energy companies, newspapers, TV, senior centers, and personal social networks provided advice regarding reducing energy use and saving money [45, 46, 52]. When faced with leaky homes, older people turned to individuals with technical expertise including engineers, builders, surveyors, and building industry authorities [66]. Some studies indicated that older adults did not have difficulties in obtaining information, but in effectively navigating, understanding, and applying it. For instance, older people in two studies expressed that the news did not effectively educate them about them about the effects of extreme temperatures on their health [43, 46]. Alternatively, other individuals were unaware of what temperatures were considered dangerous and how to efficiently heat or cool a home [44, 48, 50, 54]. In navigating online information, contracts, making use of available cost-saving initiatives, and using online banking required by some energy companies, some older people lacked the needed technical skills [41, 48, 61].

#### Recovery

Few studies examined recovery from climate and weather events, and those that did focused on handling bureaucratic reimbursement procedures, cleanup, home restoration, and securing accommodation. In two studies, older people experienced challenges in navigating post-event information concerning insurance compensation, disputes, and new building compliance requirements [59, 66]. One study described reliance on family and local communities for cleanup and repair after cyclones when public infrastructure became damaged and insurance reimbursements delayed [58]. A study from Germany mentioned a similar dependence on volunteers and community-level organizations to obtain information and help complete insurance reimbursement forms after floods [59].

Expenditure related to temporary and permanent housing after climate and weather events was addressed in several studies. One investigation reported that older adults were offered government- sponsored, temporary housing [59] while a study from Japan described increases in short-term care costs after flooding due to some families’ inability to care for senior relatives [67]. Without government assistance, older people, including those residing in multigenerational homes, were more likely to stay in place and reject relocation offers due to expensive home refinancing, unaffordable alternative energy sources, generational attachment, and a desire to preserve their identity [57, 59]. Challenges in completing repairs due to unaffordable price surges following floods was also observed [59]. Another study examining the impact of floods and cyclones in Australia found that older people were less likely to experience income loss and exhibit less severe emotional responses than younger adults [56].

## Discussion

This work represents the first systematic review to synthesize existing evidence on climate change, the care of older people, and financial expenditure. We aimed to examine the relationships between these topics and to explore the impact of climate change on spending. Across the 36 studies analyzed, which consist predominantly of evidence from high-income countries in the Global North, the literature indicates a link between climate change and financial expenditure. These costs mainly relate to health and care and temperature regulation during periods of extreme heat and cold. Through constructing a narrative based on the disaster management phases, a clear pattern emerged suggesting that several individual determinants are influenced by climate and weather exposures and also shape how older people prepare for, respond to, and recover from them. These determinants encompass behavioral, economic, environmental, social, sociodemographic, and structural attributes which likewise affect ensuing expenditure for individuals, households, public health systems, governments, and in some cases, receiving countries and residents.

Three key influences impacting preparedness, response, and recovery: support systems, access to user-friendly information, and existing economic resources.

Three interconnected themes comprising social and economic determinants appeared consistently across the preparedness, response, and recovery narrative, suggesting areas that may be particularly important for care and expenditure. The patterns observed in the included studies imply that formal and informal support systems, access to user-friendly information, and existing economic resources may shape behavior, health and care outcomes, and resulting expenditure.

First, as demonstrated in the narrative synthesis, community, family and friends, and social cohesion were attributes mentioned in the included studies that contributed to strengthening the resilience of older people. Informal support networks formed by relatives, friends, cultural affiliations, neighbors, and local communities, alongside formal support structures provided by organizations appeared to function as important social capital resources. These facilitated preparation for climate and weather impacts, adjustment to and navigation of new environments, maintenance of mental health, participation in physical activity, and the ability to trust and navigate information. Existing research from the climate change and health sectors in the Global North and South, indicate that community engagement and social support systems have a positive influence on the overall well-being of seniors [77–79]. Our analysis aligns with these findings while uncovering a more in-depth examination of specific factors influencing well-being. The synthesis of the available literature indicates that support systems play an important part in influencing behavior and expenditure and reinforces whether older people merely cope or exhibit resilience in response to extreme climate or weather exposures.

Next, an additional social determinant, access to information, appeared across all phases of the narrative. Reliance on support networks to overcome barriers in accessing information, particularly in using online services, was consistently noted in the available evidence. These barriers related to preparing for extreme weather, navigating services, identifying available opportunities for physical activity, selecting cost-effective contract options, using online banking, and completing online reimbursement procedures. Existing literature reports that this “digital divide” experienced by older people can reinforce their marginalization [80, 81]. This divide appears across several fields of influence such banking, news, and health information acquisition [82–84]. The present review expands on the economic implications of this divide in the context of climate change by highlighting a possible relationship between limited access to user-friendly information and higher individual spending. One study in this review found that some older adults were not disadvantaged in accessing government relief funds, largely due to the support they received [59]. Another study supports this finding, noting that the digital divide in developing countries is less pronounced when older people live and receive support in multi-generational households [81]. Together, these findings underscore the importance of support networks and how they can be leveraged to close gaps in accessing information. They also illustrate the importance of providing information in accessible formats to uphold equitable access to services.

Lastly, existing economic resources were the most frequently discussed determinant present in this review’s literature. Whether older people own a home, have income, savings, or insurance, may act as attributes that reinforce social inequalities relating to expenditure and care outcomes when facing climate and weather exposures. This pattern is supported by existing research indicating that older adults encounter intersecting vulnerabilities in the face of climate change, while also highlighting economic implications of these vulnerabilities [85]. For example, low income seniors using energy meters paid higher rates for fuel than those on contracts [41] and seniors living in homes with poor insulation paid more in heating costs [46, 53]. Paying higher prices because one cannot afford prepaid contracts or energy-efficient housing are examples of “poverty premiums”, or additional costs that disproportionately affect people living on limited incomes across multiple sectors. Studies report that older adults pay higher rates for utilities [86], have higher health and care costs in countries with no universal health policies [87, 88], and pay more for food due to not being able to buy in bulk. In addition to becoming increasingly marginalized, poverty premiums impact older people by creating barriers to adopting clean energy, maintaining health equity, and receiving adequate care.

### Study limitations

We only assessed studies published in English, which may have introduced representation bias in the results. As a result, evidence and narratives from the Global South are underrepresented and those from the Global North, especially from the United States and Australia, are overrepresented (see Fig. 2). This limits the generalizability of our findings to countries in the Global North and reduces transferability to Global South contexts, with different information channels, health and care systems, and fewer economic resources.

Additionally, there is a possible response bias in the data, as female participants were dominant in several qualitative studies utilizing interviews and focus groups [43, 48, 51, 55, 61, 64]. A notable limitation of this work relates to the inconsistent and broad age ranges used in several of the included studies, many of which did not stratify participants by age or reported results using large age ranges (Table 3). This made it difficult to examine age-specific differences and age trends within the evidence of the study. While older adults are considered a marginalized group, subgroups such as older women, and older individuals from racial and ethnic minorities are further underrepresented and hidden within statistics. A meta-analysis of climate and weather exposures was additionally not feasible. Inconsistent definitions of exposures such as varying criteria for heatwave duration and temperature extremes, along with subjective wording (e.g. pleasant and inclement weather) that lacked numerical data, made meaningful comparisons impracticable. Lastly, four studies were not available, which may have contributed further evidence to the narrative.

### Implications for practice, policy, and future research

Although the number of studies rose over the inclusion years, further investigation into expenditure related to preparedness and reducing response and recovery costs is needed. Based on the inclusion criteria, adults younger than 50 years were excluded. However, our analysis of the included studies suggests that several behavioral determinants (e.g. perceptions, preference, self-efficacy) influence expenditure and care outcomes. Existing evidence indicates that interventions and lifestyle changes adopted earlier in life are associated with improved long-term health outcomes, yet few studies address how decisions made across the life course shape future care needs and expenditure under climate extremes [89, 90]. Therefore, research might consider building on the current study by removing the age constraint and utilizing a life-course approach with longitudinal study design. This would facilitate investigation into how investments in preparedness and preparedness-related behaviors and choices made by young and middle-aged individuals influence future care, response, and recovery costs in older age. Furthermore, research should utilize smaller, stratified age groups that are disaggregated by subpopulations of vulnerable older adults. This would permit a better understanding of aging patterns and insight into the period at which the onset of climate and weather-related expenditures become relevant.

The observed influence of individual determinants on the resilience of older people highlights the value of conducting further investigation at sub-regional levels. For example, older adults belonging to communities with high social resilience (defined as the capacity of social groups or communities to handle and adjust to environmental, political, and social disturbances and changes) may benefit from these supportive networks during climate and weather events. Such support can translate to reduced care costs and improved quality of care [91]. Furthermore, policy makers and governments can draw upon the social cohesion of these networks to effectively communicate climate information and decision-making support materials in relevant ways that align with individuals’ attributes (e.g. in Table 5), thus closing the digital divide [92]. Policies can further work towards reducing disparities in digital access by promoting community-based training initiatives, support services that assist individuals in navigating online services, and mandates that require information on cost-effective services be communicated in several formats.

In applying a life-course approach, preventative measures can be disseminated and adopted well before mid-life. As identified in this review, temperature regulation may be attributed to additional expenditure costs, showing the need for policies that promote sustainable and cost-effective heating and cooling solutions as aging populations increase, and temperatures become more extreme. Additional funding dedicated to preventative measures could enable the retrofitting of existing housing and the construction of climate resilient buildings in locations capable of withstanding extreme climate and weather events. Health systems, care organizations, and communities would further benefit from co-designed, multisectoral heat action plans that include health and care with public services and infrastructure. Additionally, the funding of public cooling and heating centers and reimbursement schemes for energy efficient heating and cooling technologies may reduce the burden on care systems while protecting older adults from energy poverty.

A few studies excluded from this review for not fully meeting the inclusion criteria nonetheless offer valuable foundations for future research, particularly those that shed light on the domestication of informal, unpaid care. Further investigation could help quantify the indirect costs associated with unpaid care, such as opportunity costs from caregiving instead of paid employment, productivity loss as work shifts towards disaster recovery, and diminished human capital resulting from disproportionate caregiving responsibilities [93, 94]. This is especially relevant for some countries in the Global South, where great importance is placed on social values and caring for family members [95]. As policies and frameworks to mitigate climate change and alleviate the strain on health and care systems evolve, it is essential to account for the costs of care that extend beyond monetary expenses. Domestic responsibility often falls on female family and community members, highlighting critical equity issues that must be considered in research and policy. Collectively, more research is needed in Global South contexts, as these countries will experience disproportionate burden from climate change and extreme weather events while having fewer resources to respond effectively. Differences in health and care systems, governments and public service infrastructure for older adults emphasize the need for further evidence to inform more equitable preparedness, funding, and policy.

Lastly, although this review excluded articles on pollution not related to climate or weather exposures, it may be relevant to acknowledge pollution stemming from human behavior as a considerable driver of climate change. Future work could expand the scope of focus to include climate-sensitive diseases, such as dengue fever, which will increase expenditure for prevention and medical care, particularly in the Global South [96]. Additional secondary effects, like migration and resource shortages, as well as indirect costs linked to climate and weather exposures not covered in this review, such as droughts, wildfires, coastal erosion, and cascading impacts could also be investigated.

## Conclusion

This systematic review and narrative synthesis indicates that climate change, expenditure, and the care of older people is interconnected and influenced by a range of determinants. The findings reinforce the need for research and policy to uptake a life-course approach to aging and to enable people to make decisions during earlier life stages that will reinforce their ability to be resilient to climate change later in life. The available evidence on expenditure in relation to how older people react to climate and weather exposures largely focuses on the direct response to the exposure, while evidence concerning preparedness remains limited. Policymaking needs to consider the interconnected nature of the climate crisis and the implications for aging populations.

This work contributes new understanding of how support systems, accessible information, and available economic resources relate to individual behavior and spending in the context of climate and weather extremes across the preparedness, response, and recovery phases. Future research should focus on preparedness and behavior change to reduce the impact of climate or weather events on expenditure and care. The strength of this research is in its broad approach and synthesis, which presents a comprehensive range of factors and considerations affecting expenditure and health outcomes.

## Supplementary Information

Supplementary Material 1.

Supplementary Material 2.

Supplementary Material 3.

Supplementary Material 4.

Supplementary Material 5.

## Data Availability

The supplementary files contain all data analyzed in this study.

## Footnotes

1 Goal 3: Good health and well-being; Goal 7: Affordable and clean energy; Goal 10: Reduced inequalities; Goal 11: Sustainable cities and communities; Goal 13: Climate Action; Goal 17: Partnerships for the Goals.^1^
