# Supplementary material for: Climate change, trending outcomes for the care of older people, and financial expenditure: a systematic review and narrative synthesis
Source: BMC Public Health. 2026 Apr 23;26:1355. doi: 10.1186/s12889-026-27435-9 (PMC13107738; doi:10.1186/s12889-026-27435-9)
Supplement: Supplementary file 2 — Supplementary Material 2. [file 12889_2026_27435_MOESM2_ESM.pdf]

## Extended Search Strategy

| | |
|---|---|
| **PubMed**<br>https://pubmed.ncbi.nlm.nih.gov/ | ("climate chang*" OR "climate emergenc*" OR weather* OR "global warming") AND ("assisted living" OR nurs* OR facility* OR home* OR retire* OR care* OR long-term OR "aging in place") AND (cost* OR expen* OR financ* OR spend*) AND (elder* OR adult OR senior OR geriatric OR "aged care" OR "older adult*" OR "older people" OR pension*) AND ("1980/01/01"[Date - Publication] : "2023/06/28"[Date - Publication]) AND (english[Language]) NOT (preprint[Publication Type]) |
| **Scopus**<br>https://www.scopus.com/search/form.uri | TITLE-ABS-KEY("climate chang*" OR "climate emergenc*" OR weather* OR "global warming") AND TITLE-ABS-KEY("assisted living" OR nurs* OR facility* OR home* OR retire* OR care* OR "long-term" OR "aging in place") AND TITLE-ABS-KEY(cost* OR expen* OR financ* OR spend*) AND TITLE-ABS-KEY(elder* OR adult* OR senior* OR geriatric OR "aged care" OR "older adult*" OR "older people" OR pension*) AND PUBYEAR > 1979 AND PUBYEAR < 2024 AND (LIMIT-TO(LANGUAGE, "English")) AND (LIMIT-TO(SRCTYPE, "j")) AND (LIMIT-TO(DOCTYPE, "ar") OR LIMIT-TO(DOCTYPE, "re")) |
| **Web of Science**<br>https://www.webofscience.com/wos/woscc/advanced-search | TS=("climate chang*" OR "climate emergenc*" OR weather* OR "global warming") AND TS=("assisted living" OR nurs* OR facility* OR home* OR retire* OR care* OR long-term OR "aging in place") AND TS=(cost* OR expen* OR financ* OR spend*) AND TS=(elder* OR adult OR senior OR geriatric OR "aged care" OR "older adult*" OR "older people" OR pension*) AND PY=(1980-2023) AND LA=(English) NOT DT=("Preprint") |
