# Supplementary material for: Climate change, trending outcomes for the care of older people, and financial expenditure: a systematic review and narrative synthesis
Source: BMC Public Health. 2026 Apr 23;26:1355. doi: 10.1186/s12889-026-27435-9 (PMC13107738; doi:10.1186/s12889-026-27435-9)
Supplement: Supplementary file 3 — Supplementary Material 3. [file 12889_2026_27435_MOESM3_ESM.pdf]

Reasons for exclusion:

Doesnt mention expenditure (reason: cost): 169
Not related to climate change or extreme weather (reason: climate change): 52
Wrong person/age group: 119
Doesnt refer to care (reason: care): 21
no research results (reason: no results): 26
reason: no access: 4
relevant result from systematic review already included (reason: duplicate): 1

Record #	Exclusion reason	Title	Publication Year	notes:
1	climate change	"The problem is that our culture is just so messed up about aging." Recruiting older men who have sex with men (MSM) into research studies: an example from a study of aging, HIV, and anal HPV.	2022	
2	cost	24-h movement behaviour, thermal perception, thirst, and heat management strategies of children and adults during heat alerts: a pilot study	2023	
3	cost	A Case Study of a Nursing Home in Nagano, Japan: Field Survey on Thermal Comfort and Building Energy Simulation for Future Climate Change	2022	
4	cost	A Case-Only Study of Vulnerability to Heat Wave-Related Mortality in New York City (2000-2011).	2015	
5	climate change	A comparison of the thermal adaptability of people accustomed to air-conditioned environments and naturally ventilated environments.	2012	
6	wrong person group	A new mode of community continuing care service for COPD patients in China: Participation of respiratory nurse specialists	2015	
7	cost	A participatory community case study of periurban coastal flood vulnerability in southern Ecuador	2019	
8	climate change	A Patient-Centered Mobile Health System That Supports Asthma Self-Management (breathe): Design, Development, and Utilization.	2019	
9	climate change	A qualitative study on African immigrant and refugee families' experiences of accessing primary health care services in Manitoba, Canada: It's not easy!	2017	
10	wrong person group	A relationship between acute respiratory illnesses and weather.	2014	
11	cost	A retrospective study on heat-related mortality in an elderly population during the 2003 heat wave in Modena, Italy: The Argento Project	2007	
12	wrong person group	A Review of the Impact That Healthcare Risk Waste Treatment Technologies Have on the Environment	2022	
13	care	A time series study on the effects of heat on mortality and evaluation of heterogeneity into European and Eastern-Southern Mediterranean cities: Results of EU CIRCE project	2013	
14	wrong person group	A Transdisciplinary Approach to Address Climate Change Adaptation for Human Health and Well-Being in Africa.	2021	
15	climate change	Achieving Large-Scale Quality Improvement in Primary Care Annual Wellness Visits and Hierarchical Condition Coding.	2022	

16	cost	Active Living Among Older Canadians: A Time-Use Perspective Over 3 Decades	2014	
17	cost	Adverse short-term effects of ozone on cardiovascular mortalities modified by season and temperature: a time-series study.	2023	
18	cost	Aging Society and the Selected Aspects of Environmental Threats: Evidence from Poland	2020	
19	cost	Air pollution, physical activity and health: A mapping review of the evidence.	2021	
20	wrong person group	Air-quality-related health impacts from climate change and from adaptation of cooling demand for buildings in the eastern United States: An interdisciplinary modeling study.	2018	
21	cost	An analysis of the relationship between the heat index and arrivals in the emergency department	2015	
22	climate change	An individually-tailored multifactorial intervention program for older fallers in a middle-income developing country: Malaysian Falls Assessment and Intervention Trial (MyFAIT)	2014	
23	climate change	An update on sick building syndrome	2009	
24	wrong person group	An urgent call for the environmental sustainability of health systems: A ‘sextuple aim’ to care for patients, costs, providers, population equity and the planet	2023	
25	cost	Applying the marketing concept in health care: The no-show problem	1997	
26	wrong person group	Are Physicians Obliged to Lead Environmental Sustainability Efforts in Health Care Organizations?	2017	
27	wrong person group	Assessing the magnitude and uncertainties of the burden of selected diseases attributable to extreme heat and extreme precipitation under a climate change scenario in Michigan for the period 2041-2070	2019	results not stratified for age
28	no results	Assessing the nature of asthma in African epidemiological studies: a scoping review protocol	2020	protocol
29	cost	Assessment of Short- and Long-Term Mortality Displacement in Heat-Related Deaths in Brisbane, Australia, 1996-2004.	2015	
30	wrong person group	Assessment of two behavioural models (HBM and RANAS) for predicting health behaviours in response to environmental threats: Surface water flooding as a source of groundwater contamination and subsequent waterborne infection in the Republic of Ireland	2019	
31	cost	Association between ambient cold exposure and mortality risk in Shandong Province, China: Modification effect of particulate matter size.	2022	
32	cost	Association Between Atmospheric Conditions and Occurrence of Out-of-Hospital Cardiac Arrest-10-Year Population-Based Survey in Osaka	2013	
33	cost	Association between rainfall and diagnoses of joint or back pain: retrospective claims analysis.	2017	
34	climate change	Association Of A Regional Health Improvement Collaborative With Ambulatory Care-Sensitive Hospitalizations	2018	
35	cost	Association of environmental and sociodemographic factors with life satisfaction in 27 European countries.	2019	
36	cost	Associations between extreme precipitation and gastrointestinal-related hospital admissions in Chennai, India.	2014	

37	wrong person group	Associations between short-term temperature exposure and kidney-related conditions in New York State: The influence of temperature metrics across four dimensions.	2023	
39	wrong person group	Asthma control and care among six public health clinic attenders in Malaysia: A cross-sectional study.	2023	
40	cost	Barriers and facilitators to CPR knowledge transfer in an older population most likely to witness cardiac arrest: A theory-informed interview approach	2014	
41	wrong person group	Barriers to and enablers of physical activity in patients with COPD following a hospital admission: A qualitative study	2014	
42	wrong person group	Barriers to care in urban and rural dwelling adults with congenital heart disease	2022	
43	wrong person group	Barriers to Physical Activity Among African American Women: An Integrative Review of the Literature.	2015	
44	care	Benefits of Mobile Phone Technology for Personal Environmental Monitoring	2016	not relevant to research question
45	cost	Beyond Usual Geographical Scales of Analysis: Implications for Healthcare Management and Urban Planning	2022	
46	cost	Brachial artery responses to ambient pollution, temperature, and humidity in people with type 2 diabetes: a repeated-measures study.	2014	
47	wrong person group	Budgeting for climate change: obstacles and opportunities at the US state level	2018	
48	no results	Building a more sustainable future for senior living	2011	
49	wrong person group	Building resilience to climate risks through social protection: from individualised models to systemic transformation.	2019	
50	cost	Burden and risk factors for cold-related illness and death in New York city	2018	
51	cost	Burden of Outpatient Visits Attributable to Ambient Temperature in Qingdao, China.	2021	
52	no results	Call for Emergency Action to Limit Global Temperature Increases, Restore Biodiversity, and Protect Health.	2021	call for action
53	cost	Can pervasive sensing address current challenges in global healthcare?	2012	
54	no results	Can we help the losers in climate change?	2016	website statement? No doi
55	wrong person group	Carbon footprint and associated costs of asthma exacerbation care among UK adults	2022	
56	cost	Characteristics and Circumstances of Falls in the Community-Dwelling Older Adult Population	2020	
57	wrong person group	Characteristics Associated With Unsheltered Status Among Veterans	2021	
58	cost	Climate Change Adaptation: Prehospital Data Facilitate the Detection of Acute Heat Illness in India.	2021	
59	wrong person group	Climate change and credit risk	2020	
60	no results	Climate Change and Health: A Position Paper of the American College of Physicians	2016	position paper
61	wrong person group	Climate change and health in Bangladesh: A baseline cross-sectional survey	2016	avg age 44.7

62	cost	Climate change and health in the Mid-Atlantic Region	2000	mentioned but not tied to age
63	no results	Climate Change and Health in the Urban Environment: Adaptation Opportunities in Australian Cities	2011	not a research paper
64	cost	Climate Change and Health: Consequences of High Temperatures among Vulnerable Groups in Finland	2022	
65	cost	Climate change and health: Indoor heat exposure in vulnerable populations	2012	
66	care	Climate change and the fiduciary duties of pension fund trustees–lessons from the Australian law	2016	
67	no results	Climate change and the public health imperative for supporting migration as adaptation.	2023	charter
68	cost	Climate change vulnerability and social development for remote indigenous communities of South Australia	2012	Says elders were asked, however results dont refer specifically to elderly care, general comments about prices increasing
69	wrong person group	Climate change: Challenges and opportunities for global health	2014	
70	cost	Climatic burden of eating at home against away-from-home: A novel Bayesian Belief Network model for the mechanism of eating-out in urban China	2019	
71	climate change	Clinical Outcomes, Costs, and Cost-effectiveness of Strategies for Adults Experiencing Sheltered Homelessness During the COVID-19 Pandemic	2020	
72	climate change	Clinician Perspectives on Cannulation for Extracorporeal Cardiopulmonary Resuscitation: A Mixed Methods Analysis	2023	
73	cost	Coastal flooding and frontline health care services: challenges for flood risk resilience in the English health care system	2019	some results about seniors, costs not specific to seniors
74	wrong person group	Cold homes and mental health harm: Evidence from the UK Household Longitudinal Study	2022	
75	wrong person group	Combating climate change in the clinic: Cost-effective strategies to decrease the carbon footprint of outpatient dermatologic practice	2021	

77	cost	Common values in assessing health outcomes from disease and injury: disability weights measurement study for the Global Burden of Disease Study 2010	2012	
78	wrong person group	Community acceptance challenges of renewable energy transition: A tale of two solar parks in Limpopo, South Africa	2018	
79	climate change	Community-dwelling older adults who are low-income and disabled weathering financial challenges	2021	
80	cost	Comparison of Summer and Winter Objectively Measured Physical Activity and Sedentary Behavior in Older Adults: Age, Gene/Environment Susceptibility Reykjavik Study	2017	
81	climate change	Conceptual Relationship Between Traditional Persian Medicine and Modern Nutrition in Middle Age Obesity	2022	
82	climate change	Connecting the Dots between Social Care and Healthcare for the Sustainability Development of Older Adult in Asia: A Scoping Review	2022	
84	care	Coronavirus comes home? Energy use, home energy management, and the social-psychological factors of COVID-19.	2020	
85	climate change	Cost effectiveness of preventive home visits to the elderly: Economic evaluation alongside randomized controlled study	2006	
86	wrong person group	Cost-effectiveness analysis of the New South Wales adult drug court program.	2004	
87	wrong person group	COVID-19 Solutions Are Climate Solutions: Lessons From Reusable Gowns.	2020	
89	cost	Dehydration and renal failure in older persons during heatwaves-predictable, hard to identify but preventable?	2019	
90	climate change	Demand response to improved walking infrastructure: A study into the economics of walking and health behaviour change	2015	
91	cost	Design strategies for houses subject to heatwaves	2012	
92	cost	Determinants of dietary behavior and physical activity among Canadian Inuit: a systematic review	2015	
93	cost	Determining wellness through an ambient assisted living environment	2014	
94	climate change	Developing English domestic occupancy profiles	2019	
95	wrong person group	Development and Validation of the Return and Disposal of Unused Medications Questionnaire (ReDiUM) in Malaysia	2018	
96	wrong person group	Diagnostic of the massive flood event and flood hazard mapping in Tons River basin	2022	
97	no results	Diaspora, disease, and health care	2007	not a research paper
98	climate change	Did retirees save enough to compensate for the increase in individual risk exposure?	2010	
99	cost	Disability-adjusted life years (DALYs) for 291 diseases and injuries in 21 regions, 1990-2010: a systematic analysis for the Global Burden of Disease Study 2010	2012	
100	no access	Distribution features of the weather conditions in Nha Trang Bay (The South China Sea)	2022	
101	cost	Do catastrophic floods change the gender division of labor? Panel data evidence from Pakistan	2021	
102	wrong person group	Do domestic heating controls save energy? A review of the evidence	2018	

103	wrong person group	Do the COVID-19 Crisis, Ageing and Climate Change Put Swiss Fiscal Sustainability at Risk?	2022	
104	cost	Does poverty reduce mental health? An instrumental variable analysis	2014	
105	cost	Drought and all-cause mortality in Nebraska from 1980 to 2014: Time-series analyses by age, sex, race, urbanicity and drought severity.	2022	
106	cost	Economic and mental health impacts of multiple adverse events: Hurricane Harvey, other flooding events, and the COVID-19 pandemic	2022	
107	climate change	Economic burden of dengue fever in China: A retrospective research study	2022	
109	care	Effect of axial heat transfer and atmospheric conditions on the energy performance of GSHP systems: A simulation-based analysis	2015	
110	cost	Effective Community-Based Interventions for the Prevention and Management of Heat-Related Illnesses: A Scoping Review	2021	
111	cost	Effectiveness of Public Health Interventions in Reducing Morbidity and Mortality during Heat Episodes: a Structured Review	2010	
112	cost	Effects of 2018 Japan floods on healthcare costs and service utilization in Japan: a retrospective cohort study	2023	
113	wrong person group	Effects of ambient temperature on atopic dermatitis and attributable health burden: a 6-year time-series study in Chengdu, China.	2023	
114	cost	Effects of Environmental and Socioeconomic Inequalities on Health Outcomes: A Multi-Region Time-Series Study	2022	
115	cost	Effects of heat wave on body temperature and blood pressure in the poor and elderly.	2012	
116	cost	Effects of mechanical ventilation on indoor air quality and occupant health status in energy-efficient homes: A longitudinal field study.	2021	
117	cost	Effects of the nursing intervention Fall prevention in older adults with arterial hypertension using NANDA-I, NIC, and NOC	2022	
119	cost	Emergency Visits for Thunderstorm-Related Respiratory Illnesses Among Older Adults.	2020	
120	care	Emerging fiscal health and governance concerns resulting from COVID-19 challenges	2021	
121	wrong person group	Enablers and barriers to effective diabetes self-management: A multi-national investigation	2019	
123	care	Energy poverty: an overlooked determinant of health and climate resilience in Canada	2023	
124	climate change	Enhancing efficiency and quality of ambulatory care through telehealth technology.	2005	
125	no results	Environmental health advancing emancipatory policies for the common good	2018	
126	wrong person group	Environmental impact of cardiovascular healthcare	2023	
127	climate change	Environmental impact of meal service catering for dependent senior citizens in Danish municipalities	2019	pollution
128	no results	Environmental stewardship and healthcare: global reflections for radiology	2022	statement, reflection
129	wrong person group	Environmental stressors and cardio-metabolic disease: part I-epidemiologic evidence supporting a role for noise and air pollution and effects of mitigation strategies.	2017	

130	cost	Environmental Sustainability in Respiratory Care: An Overview of the healthCARe-Based envirONmental Cost of Treatment (CARBON) Programme	2022	
131	cost	Epidemiologic Trends and Seasonality of Scabies in South Korea, 2010-2017	2019	
132	cost	Epidemiological, socio-demographic and clinical features of the early phase of the COVID-19 epidemic in Ecuador.	2021	
133	cost	Epidemiology of aspergillosis in hospitalised Spanish patients—A 21-year retrospective study	2021	
134	climate change	Estimating cardiovascular hospitalizations and associated expenses attributable to ambient carbon monoxide in Lanzhou, China: Scientific evidence for policy making	2019	air pollution
135	cost	Estimating the burden of heat illness in England during the 2013 summer heatwave using syndromic surveillance	2016	
136	wrong person group	Estimating Willingness to Pay for Online Health Services with Discrete-Choice Experiments.	2017	
137	cost	Estimation of effects of extreme temperature on the risk of hospitalisation in Taiwan	2023	
138	climate change	Estimation of the Effects of Air Pollution on Hospitalization Expenditures for Asthma	2020	air pollution
140	no results	Evaluating a Long-Term Livable Communities Strategy in the U.S.	2011	recommendations
141	care	Evidence for lower plasticity in CTMAX at warmer developmental temperatures	2018	
142	no results	Exacerbation-Prone Asthma: A Biological Phenotype or a Social Construct	2021	statement
143	wrong person group	Examining Facilitators and Barriers to Cardiac Rehabilitation Adherence in a Low-Resource Setting in Latin America from Multiple Perspectives	2022	not able to separate results to aged 50+
145	cost	Examining the magnitude and perception of summertime overheating in London care homes	2021	
146	cost	Examining the Walking Accessibility, Willingness, and Travel Conditions of Residents in Saudi Cities	2019	
147	cost	Excess hospital admissions for pneumonia, chronic obstructive pulmonary disease, and heart failure during influenza seasons in Hong Kong	2004	
148	care	Exercise-based transportation reduces oil dependence, carbon emissions and obesity	2005	
149	climate change	Expanding access to health care for Social Security Disability Insurance beneficiaries: early findings from the accelerated benefits demonstration.	2010	
151	wrong person group	Exploring chronic airways disease patients' perspectives on self-management topics.	2022	
152	cost	Exploring the Spatial Distribution of Rheumatic Diseases and Its Correlation With Temperature and Humidity Among Middle-Aged and Elderly Adults in China	2022	
153	wrong person group	Exposure to Greenness and Mortality in a Nationwide Prospective Cohort Study of Women.	2016	
156	climate change	Factors associated with costs and health outcomes in patients with Back and leg pain in primary care: a prospective cohort analysis	2019	
157	cost	Factors Associated With Poor Health Outcomes Among Vietnamese Americans After Hurricane Harvey, Houston, Texas, 2018-2019	2023	

158	wrong person group	Field interventions for climate change mitigation behaviors: A second-order meta-analysis	2023	
159	no results	Financial Planning for Retirement: A Psychosocial Perspective.	2017	perspective article
160	wrong person group	First Nations households living on-reserve experience food insecurity: prevalence and predictors among ninety-two First Nations communities across Canada	2021	
161	cost	Flood survivors' perspectives on vulnerability reduction to floods in Mbire district, Zimbabwe	2020	
162	no results	FOODLIT-Trial: Protocol of a Randomised Controlled Digital Intervention to Promote Food Literacy and Sustainability Behaviours in Adults Using the Health Action Process Approach and the Behaviour Change Techniques Taxonomy during the COVID-19 Pandemic	2022	protocol
163	wrong person group	Forced displacements and the environment: Its place in national and international climate agenda	2018	
164	wrong person group	Fresh driver for economic growth: fracking the UK nation	2015	
165	wrong person group	Fuel poverty and human health: A review of recent evidence	2010	
166	wrong person group	Gender-based experiences and perceptions after the 2010 winter storms in Atlantic Canada	2015	
167	cost	General Practitioners' Perceptions of Heat Health Impacts on the Elderly in the Face of Climate ChangeA Qualitative Study in Baden-Wurttemberg, Germany	2018	
168	cost	Geomagnetic disturbances reduce heart rate variability in the Normative Aging Study.	2022	
169	no results	Geriatric medicine in the era of climate change	2022	research article- not a study
170	no results	Global Warming in Pakistan and Its Impact on Public Health as Viewed Through a Health Equity Lens.	2023	research article- not a study
171	cost	Global, regional, and national burden of stroke and its risk factors, 1990-2019: a systematic analysis for the Global Burden of Disease Study 2019	2021	
172	cost	Global, regional, and national incidence, prevalence, and years lived with disability for 328 diseases and injuries for 195 countries, 1990-2016: a systematic analysis for the Global Burden of Disease Study 2016	2017	
173	cost	Global, regional, and national life expectancy, all-cause mortality, and cause-specific mortality for 249 causes of death, 1980-2015: a systematic analysis for the Global Burden of Disease Study 2015	2016	
174	climate change	Goal setting and strategies to enhance goal pursuit for adults with acquired disability participating in rehabilitation.	2015	
175	cost	Green Space, Air Pollution, Weather, and Cognitive Function in Middle and Old Age in China	2022	
176	climate change	Health beliefs and patient perspectives of febrile illness in Kilombero, Tanzania	2019	
177	wrong person group	Health care facilities resilient to climate change impacts	2014	
178	wrong person group	Health coping strategies of the people vulnerable to climate change in a resource-poor rural setting in Bangladesh	2013	

179	wrong person group	Health gender gap in Uganda: do weather effects and water play a role?	2022	results not stratified for age
180	wrong person group	Health impact assessment by the implementation of Madrid City air-quality plan in 2020.	2020	
181	wrong person group	Health promotores' perceptions of their communities' health needs, knowledge, and resource needs in rural Nicaragua.	2013	
182	wrong person group	Healthy and climate-friendly eating patterns in the New Zealand context	2020	
183	wrong person group	Healthy dietary choices and physical activity participation in the canadian arctic: Understanding nunavut inuit perspectives on the barriers and enablers	2021	
184	cost	Heat Emergencies: Perceptions and Practices of Community Members and Emergency Department Healthcare Providers in Karachi, Pakistan: A Qualitative Study	2021	
185	cost	Heat Exposure Following the Rainy Season Is Associated With an Increased Risk of Cardiovascular Emergency Among the Elderly in Japan	2023	
186	wrong person group	Heat Illness Requiring Emergency Care for People Experiencing Homelessness: A Case Study Series	2022	
187	wrong person group	Heat or Eat? Cold-Weather Shocks and Nutrition in Poor American Families	2003	
188	wrong person group	Heat wave adaptations for UK dwellings and development of a retrofit toolkit	2013	
189	cost	Heat wave impact on morbidity and mortality in the elderly population: a review of recent studies	2011	
190	cost	Heat-related first cardiovascular event incidence in the city of Madrid (Spain): Vulnerability assessment by demographic, socioeconomic, and health indicators	2023	
192	cost	Heatwaves, hospitals and health system resilience in England: a qualitative assessment of frontline perspectives from the hot summer of 2019	2023	
193	cost	High Temperature and Hospitalizations for Cardiovascular and Respiratory Causes in 12 European Cities	2009	
194	cost	High temperatures and emergency department visits in 18 sites with different climatic characteristics in China: Risk assessment and attributable fraction identification	2020	
195	wrong person group	Hot temperatures can force delayed mosquito outbreaks via sequential changes in Aedes aegypti demographic parameters in autocorrelated environments	2014	
196	wrong person group	Houston hurricane Harvey health (Houston-3H) study: assessment of allergic symptoms and stress after hurricane Harvey flooding.	2021	
197	climate change	How to Obtain Forty Percent Less Environmental Impact by Healthy, Protein-Optimized Snacks for Older Adults	2017	
198	cost	Human health outcomes at the neighbourhood scale implications: Elderly's heat-related cardiorespiratory mortality and its influencing factors.	2021	
199	no results	Hydration, morbidity, and mortality in vulnerable populations	2012	
200	wrong person group	Hydroclimate changes in eastern Africa over the past 200,000 years may have influenced early human dispersal	2021	
201	care	Hydro-diplomacy: Opportunities for learning from an interregional process	2006	

202	no results	Identifying Linkages Between Climate Change, Urbanisation, and Population Ageing for Understanding Vulnerability and Risk to Older People: A Review	2022	review article
203	wrong person group	Identifying similar and different factors effecting long-term cardiac exercise rehabilitation behavior modification between New Zealand and the United Kingdom.	2014	
204	cost	Identifying the attentional demands perceived by elderly people.	1998	
205	wrong person group	III. Factors affecting emergency service utilization at a rural community mental health center	1998	
206	wrong person group	Impact of 2014 Kashmir flood on land use/land cover transformation in Dal lake and its surroundings, Kashmir valley	2020	
207	climate change	Impact of an innovative financing and payment model on tuberculosis patients' financial burden: Is tuberculosis care more affordable for the poor?	2019	
208	cost	Impact of short-term exposure to extreme temperatures on diabetes mellitus morbidity and mortality? A systematic review and meta-analysis.	2021	
209	cost	Impacts of heat waves and corresponding measures: a review	2015	
211	cost	Impacts on human health in the Arctic owing to climate-induced changes in contaminant cycling - The EU ArcRisk project policy outcome	2015	
212	cost	Improved indoor air quality during desert dust storms: The impact of the MEDEA exposure-reduction strategies.	2023	
213	no results	Improving kidney care for residents in nursing facilities: a national model.	2016	
214	care	Income, housing and health: Poverty in the United States through the prism of residential energy efficiency programs	2021	
215	wrong person group	Increasing impacts of temperature on hospital admissions, length of stay, and related healthcare costs in the context of climate change in Adelaide, South Australia.	2021	
216	cost	Independent effect of weather, air pollutants, and seasonal influenza on risk of tuberculosis hospitalization: An analysis of 22-year hospital admission data.	2022	
217	cost	Indirect consequences of extreme weather and climate events and their associations with physical health in coastal Bangladesh: a cross-sectional study	2015	
218	no results	Individual, social and physical environmental correlates of sedentary behaviours in adults: A systematic review protocol	2014	protocol for systematic review
219	wrong person group	Indoor temperatures in low cost housing in Johannesburg, South Africa	2017	
220	cost	Influence of climate on emergency department visits for syncope: role of air temperature variability	2011	
221	cost	Influence of seasonal variations on physical activity in older people living in mountainous agricultural areas.	2019	
222	care	Influences of extreme weather, climate and pesticide use on invertebrates in cereal fields over 42 years	2015	
223	cost	Influential factors of age-friendly neighborhood open space under high-density high-rise housing context in hot weather: A case study of public housing in Hong Kong	2021	

224	no results	Informed decision-making in delivery of dialysis: Combining clinical outcomes with sustainability	2021	
225	cost	Innovation in Healthy and Sustainable Food Product Development for Health and Aged Care: A Scoping Review	2022	
226	wrong person group	Insurance pricing for windstorm-susceptible developments: Bootstrapping approach	2012	
227	wrong person group	Integrating drones into NHS patient diagnostic logistics systems: Flight or fantasy?	2022	
228	climate change	Integrating funds for health and social care: an evidence review.	2015	
229	wrong person group	Investigating Indian summer monsoon in coupled regional land-atmosphere downscaling experiments using RegCM4	2020	
230	climate change	Investigating the economic case of a service to support carers of people with dementia: A cross-sectional survey-based feasibility study in England.	2019	
231	wrong person group	Kenyan women bearing the cost of climate change	2021	
232	cost	Knowledge and perception about climate change and human health: Findings from a baseline survey among vulnerable communities in Bangladesh	2016	
233	wrong person group	Knowledge co-production for Indigenous adaptation pathways: Transform post-colonial articulation complexes to empower local decision-making	2020	
234	climate change	Linking widowhood and later-life depressive symptoms: Do childhood socioeconomic circumstances matter?	2022	
235	care	Living at High Altitude and COVID-19 Mortality in Peru.	2022	
237	no access	Long-term care alternatives in northern communities.	1981	
238	care	Long-Term Impacts of Diurnal Temperature Range on Mortality and Cardiovascular Disease: A Nationwide Prospective Cohort Study.	2022	
239	cost	Long-term ozone exposure and cognitive impairment among Chinese older adults: A cohort study.	2022	
240	cost	Long-term residential sunlight exposure associated with cognitive function among adults residing in Finland	2022	
241	cost	Long-term temperature variability and the incidence of cardiovascular diseases: A large, representative cohort study in China.	2021	
243	wrong person group	Managing a sustainable, low carbon supply chain in the English National Health Service: The views of senior managers	2013	
244	cost	Managing the health effects of climate change. Lancet and University College London Institute for Global Health Commission	2009	
245	wrong person group	Mapping development and health effects of cooking with solid fuels in low-income and middle-income countries, 2000-18: a geospatial modelling study.	2022	
246	cost	Measuring the burden of disease due to climate change and developing a forecast model in South Korea.	2014	
247	cost	Measuring the environmental burden of disease in South Korea: A population-based study	2015	
248	climate change	Medicaid Expansion: Effects On Hospital Finances And Implications For Hospitals Facing COVID-19 Challenges.	2021	

249	cost	Meeting Vitamin D Requirements in White Caucasians at UK Latitudes: Providing a Choice	2018	
250	wrong person group	Meteorological parameters and hospitalizations of patients with sickle cell anemia: a 20-year retrospective study in Campinas, Sao Paulo, Brazil	2022	
251	cost	Mind the Gap: Built Infrastructures, Sustainable Caring Relations, and Resilient Communities in Extreme Weather Events	2013	
252	cost	Motivating metrics for household water-use feedback	2015	
253	cost	Motivations, barriers, and strategies for meat reduction at different family lifecycle stages.	2020	
255	cost	Neighborhood effects on heat deaths: social and environmental predictors of vulnerability in Maricopa County, Arizona.	2013	
256	no access	New approaches for the elderly show significant results.	1999	
257	cost	Night-time activity forecast by season and weather in a longitudinal design - natural light effects on three years' rest-activity cycles in nursing home residents with dementia	2017	
258	wrong person group	No seasonal variation in physical activity of Han Chinese living in Beijing	2017	
259	no results	Non-communicable disease epidemic: epidemiology in action (EuroEpi 2013 and NordicEpi 2013): Aarhus, Denmark from 11 August to 14 August 2013	2013	abstract
260	cost	Novel therapeutic concepts: The epidemic of cardiovascular disease in the developing world: Global implications	2010	
261	cost	Nutrition leadership training in North-East Asia: an IUNS initiative in conjunction with nutrition societies in the region.	2008	
262	cost	Occupant satisfaction with indoor environmental quality and health after energy retrofits of multi-family buildings: Results from INSULAtE-project	2018	
263	wrong person group	Occupational heat stress and economic burden: A review of global evidence	2021	
265	no access	Older people still struggling to keep warm, figures show.	2005	
266	care	On the association of lightning activity and projected change in climate over the Indian sub-continent	2017	
267	climate change	Optimal Asthma Control: Time for a New Target.	2020	
268	climate change	Outcomes of reablement and their measurement: Findings from an evaluation of English reablement services.	2019	
269	wrong person group	Pacific island health inequities forecast to grow unless profound changes are made to health systems in the region.	2017	
270	cost	Peace of mind: A community-industry-academic partnership to adapt dementia technology for Anishinaabe communities on Manitoulin Island	2020	
271	climate change	Pension systems and the crisis: Weathering the storm	2010	
272	wrong person group	Perceived Health Impacts, Sources of Information and Individual Actions to Address Air Quality in Two Cities in Nigeria	2023	
274	wrong person group	Perceptions of participants and staff of implementing a physical activity program in rural primary care.	2020	
275	wrong person group	Performance and implementation of low impact development - A review	2017	

276	wrong person group	Personal cooling with phase change materials to improve thermal comfort from a heat wave perspective	2012	
277	wrong person group	Personal values, beliefs, and ecological risk perception.	2006	
278	climate change	Personalized implementation of video telehealth for rural veterans (PIVOT-R)	2021	
279	wrong person group	Physical activity among Somali men in Minnesota: barriers, facilitators, and recommendations.	2014	
280	cost	Physical environment research of the family ward for a healthy residential environment.	2022	
281	wrong person group	Piloting a low-cost hardware intervention to reduce improper disposal of solid waste in communal toilets in low-income settlements in Dhaka, Bangladesh	2017	
282	climate change	Planned Retirement Timing in Europe: Are Europeans Adapting to the Policy of Extending Working Lives.	2021	
283	climate change	Population Aging Driven Slowdown in the Reduction of Economic Cost-Attributed to PM(2.5) Pollution after 2013 in China.	2022	
284	climate change	Power to the patient.	1997	
285	cost	Precipitation Increases the Occurrence of Sporadic Legionnaires' Disease in Taiwan	2014	
286	care	Predicted temperature-increase-induced global health burden and its regional variability	2019	
287	climate change	Predicting excess cost for older inpatients with clinical complexity: A retrospective cohort study examining cognition, comorbidities and complications.	2018	
288	cost	Pre-earthquake burden of illness and postearthquake health and preparedness in veterans.	2014	
289	wrong person group	Preparing for an epidemic of limited health literacy: Weathering the perfect storm	2008	
290	wrong person group	Prevalence and severity of household food insecurity of First Nations people living in an on- reserve, sub- Arctic community within the Mushkegowuk Territory	2014	
291	cost	Prevalence of a Healthy Lifestyle Among Individuals With Cardiovascular Disease in High-, Middle- and Low-Income Countries The Prospective Urban Rural Epidemiology (PURE) Study	2013	
293	climate change	Projecting social support needs of informal caregivers in Malaysia.	2014	
294	cost	Projection of future hot weather events and potential population exposure to this in South Korea	2017	
295	wrong person group	Promoting environmental sustainability, equity and health in Victorian Primary Care Partnerships	2020	
296	wrong person group	Psychosocial stress and strategies for managing adversity: Measuring population resilience in New South Wales, Australia	2010	
297	wrong person group	Public health and natural hazards: new policies and preparedness initiatives developed from an Australian bushfire case study	2019	
298	cost	Public Health Aspects of Climate Change Adaptation in Three Cities: A Qualitative Study	2022	
299	cost	Public Perceptions of Climate Change and Health-A Cross-Sectional Survey Study.	2023	
301	wrong person group	Quantifying hospital services by carbon footprint: A systematic literature review of patient care alternatives	2020	
302	no results	Reablement services for people at risk of needing social care: the MoRe mixed-methods evaluation	2019	not peer reviewed
303	cost	Reflections on Cross-Cultural Comparison of the Impact of Housing Modification/Adaptation* for Supporting Older People at Home: A Discussion	2020	

304	wrong person group	Regulation to Create Environments Conducive to Physical Activity: Understanding the Barriers and Facilitators at the Australian State Government Level	2012	
305	cost	Relationship between climate and psychiatric inpatient length of stay in Veterans Health Administration hospitals.	2000	
306	cost	Relationship between meteorological variations, seasonal influenza, and hip fractures in the elderly: A modelling investigation using 22-year data.	2023	
307	cost	Relationships between home ventilation rates and respiratory health in the Colorado Home Energy Efficiency and Respiratory Health (CHEER) study	2019	
308	wrong person group	Relationships between Long-Term Demography and Weather in a Sub-Arctic Population of Common Eider	2013	
309	no results	Report of the Lancet Commission on the Value of Death: bringing death back into life.	2022	
310	care	Residents' lamp purchasing behaviour, indoor lighting characteristics and choices in Swedish homes	2019	
311	cost	Respiratory Effects of Indoor Heat and the Interaction with Air Pollution in Chronic Obstructive Pulmonary Disease.	2016	
313	climate change	Risk factors of multiple sclerosis and their relation with disease severity: A cross-sectional study from Iran	2016	
314	wrong person group	Risk factors precipitating exacerbations in adult asthma patients presenting at Kalafong Hospital, Pretoria	2008	
315	cost	Risk of Fall-Related Injury due to Adverse Weather Events, Philadelphia, Pennsylvania, 2006-2011	2017	
316	wrong person group	Safety nets can help address the risks to nutrition from increasing climate variability.	2010	
317	cost	Same pollution sources for climate change might be hyperactivating the NLRP3 inflammasome and exacerbating neuroinflammation and SARS mortality.	2021	
319	cost	Seasonal variations in objectively assessed physical activity among people with COPD in two Nordic countries and Australia: a cross-sectional study.	2019	
321	cost	Seasonality in physical activity and walking of healthy older adults	2015	
322	wrong person group	Seasonality, household food security, and nutritional status in Dinajpur, Bangladesh.	2008	
323	cost	Seasonally Dependent Change of the Number of Fractures after 50 Years of Age in Poland-Analysis of Combined Health Care and Climate Datasets	2022	
324	climate change	Secondary data analysis of intertrigo in hospital and geriatric settings: a comparison of prevalence, anatomical locations, and interventions.	2022	
325	cost	Self-esteem and communal responsiveness toward a flawed partner: the fair-weather care of low-self-esteem individuals.	2009	
326	wrong person group	Shared decision making: Does a physician's decision-making style affect patient participation in treatment choices for primary immunodeficiency?	2019	
327	cost	Short-term effects of ambient air pollution on chronic obstructive pulmonary disease admissions in Beijing, China (2013-2017).	2019	
330	cost	Sociodemographic factors associated with time to discharge for hospitalised patients with asthma and asthma exacerbation using the Ghana Health Service District Information Management System 2 (DHIMS-2) database, 2012-2017	2021	

331	cost	Solidarity and care as relational practices	2018	
332	wrong person group	Solidarity and the problem of structural injustice in healthcare	2018	
333	cost	Source apportionment of indoor PM10 in Elderly Care Centre.	2016	
334	cost	Staying cool in a changing climate: Reaching vulnerable populations during heat events.	2013	
335	cost	Stormy weather: a retrospective analysis of demand for emergency medical services during epidemic thunderstorm asthma.	2017	
336	cost	Street temperature and building characteristics as determinants of indoor heat exposure.	2021	
337	cost	Summer indoor heat exposure and respiratory and cardiovascular distress calls in New York City, NY, U.S.	2016	
339	cost	Summer thermal comfort and overheating in the elderly	2019	
340	cost	Summertime thermal conditions and senior resident behaviors in public housing: A case study in Elizabeth, NJ, USA	2020	
341	cost	Survey of county-level heat preparedness and response to the 2011 summer heat in 30 U.S. States	2014	
342	cost	Susceptibility of the Aging Lung to Environmental Injury	2010	
343	cost	Sustainable healthcare - Time for 'Green Podiatry'.	2021	
344	cost	Systematic causality mapping of factors leading to accidental falls of older adults.	2020	
345	cost	Talking about Climate Change and Environmental Degradation with Patients in Primary Care: A Cross-Sectional Survey on Knowledge, Potential Domains of Action and Points of View of General Practitioners	2022	
346	climate change	Telehealth and the global health network in the 21st century. From homecare to public health informatics.	2001	
347	no results	Telemedicine for Patient Management in Remote Areas and Underserved Populations	2023	presentation
348	climate change	Tell someone who cares': Participation action research on workplace engagement of caregivers in aged residential care, NZ.	2021	
349	cost	Temperature and non-communicable diseases: Evidence from Indonesia's primary health care system	2022	
351	cost	Temperature variability and common diseases of the elderly in China: a national cross-sectional study.	2023	
352	cost	Temperature variability increases the onset risk of ischemic stroke: A 10-year study in Tianjin, China.	2023	
353	cost	Temperature-related chest pain presentations and future projections with climate change.	2022	
355	wrong person group	The avoidable health effects of air pollution in three Latin American cities: Santiago, São Paulo, and Mexico City	2006	
356	cost	The burden of air pollution on years of life lost in Beijing, China, 2004-08: retrospective regression analysis of daily deaths.	2013	
357	wrong person group	The Changes in Climate Change Concern, Responsibility Assumption and Impact on Climate-friendly Behaviour in EU from the Paris Agreement Until 2019	2022	
358	climate change	The changes of activity-travel participation across gender, life-cycle, and generations in Sweden over 30 years	2019	
359	cost	The changing health needs of the UK population	2021	
360	cost	The consequences for human health of stratospheric ozone depletion in association with other environmental factors.	2015	

362	climate change	The cultural and structural motivations of cheap mobility: The case of retirement migrants in Spain and Costa Rica	2021	relates to pollution
363	cost	The determinants of COVID-19 case fatality rate (CFR) in the Italian regions and provinces: An analysis of environmental, demographic, and healthcare factors.	2021	costs not stratified for age
364	climate change	The effect of assigning dedicated general practitioners to nursing homes.	2019	
365	cost	The effect of extreme cold temperatures on the risk of death in the two major Portuguese cities	2017	
366	wrong person group	The effect of global change on mosquito-borne disease	2019	
367	cost	The effect of the Winter Fuel Payment on household temperature and health: a regression discontinuity design study	2019	
368	climate change	The effectiveness and cost-effectiveness of respite for caregivers of frail older people.	2007	
369	cost	The effects of racism, social exclusion, and discrimination on achieving universal safe water and sanitation in high-income countries	2023	
370	cost	The effects of weather on daily emergency ambulance service demand in Taipei: a comparison with Hong Kong.	2020	
371	wrong person group	The enablers, barriers and preferences of accessing radiation therapy facilities in the rural developed world - a systematic review	2017	
372	wrong person group	The environmental impact of inhaler replacement: A carbon footprint and economic calculation of the National Database of Health Insurance Claims in Japan	2023	
373	care	The Extinction of Dengue through Natural Vulnerability of Its Vectors	2010	
374	climate change	The future of death in America.	2011	
375	cost	The future of geriatric medicine	2012	not tied to CC or care
376	cost	The future? Let’s first write a narrative of our present	2020	
377	wrong person group	The global burden of kidney disease and the sustainable development goals	2018	
378	wrong person group	The impact of early-life shocks on adult welfare in Brazil: Questions of measurement and timing	2020	
379	wrong person group	The Impact of Environmental Factors on Credit Ratings in Healthcare.	2022	
380	cost	The Impact of Extreme Temperature Shocks on the Health Status of the Elderly in China.	2022	
381	cost	The influence of heatwave temperatures on fracture patient presentation to hospital.	2022	
382	wrong person group	The influence of individualistic worldviews on severe weather preparation	2022	
383	cost	The Influence of Weather Conditions on Joint Pain in Older People with Osteoarthritis: Results from the European Project on OSteoArthritis	2015	
384	wrong person group	The interaction between physical and social-psychological factors in indoor environmental health	2003	
386	cost	The Lancet Commission on pollution and health	2018	
387	wrong person group	The limits of demographic buffering in coping with environmental variation	2021	

388	no results	The process of purchasing a motorized health vehicle.	2012	description of a process
389	cost	The role of extreme high humidex in depression in chongqing, China: A time series-analysis.	2023	
390	cost	The role of plant-based alternative foods in sustainable and healthy food systems: Consumption trends in the UK.	2022	
391	climate change	The social and family dynamics behind the uptake of cataract surgery: findings from Kilimanjaro region, Tanzania.	2005	
394	cost	The Tsinghua–Lancet Commission on Healthy Cities in China: unlocking the power of cities for a healthy China	2018	
395	cost	Thematic analysis of intensive care unit diaries kept by staff: insights for caring.	2023	
396	wrong person group	There's nowhere to go: counting the costs of extreme weather to the homeless community.	2019	
397	wrong person group	Thermal strategies vary with life history stage.	2018	
398	cost	Time course of blood oxygen saturation responding to short-term fine particulate matter among elderly healthy subjects and patients with chronic obstructive pulmonary disease.	2020	
399	cost	Time Out-of-Home and Cognitive, Physical, and Emotional Wellbeing of Older Adults: A Longitudinal Mixed Effects Model	2015	
400	wrong person group	To refer or not to refer: A qualitative study of reasons for referral from role 1	2012	
401	wrong person group	Turning green: The impact of changing to more eco-friendly respiratory healthcare-a carbon and cost analysis of Dutch prescription data	2022	
402	wrong person group	Type 2 Diabetes Patients' Perspectives, Experiences, and Barriers Toward Diabetes-Related Self-Care: A Qualitative Study From Pakistan	2020	
403	wrong person group	Uncovering urban residents’ electricity conservation and carbon reduction potentials in megacities of China–A systematic path of behavioural interventions	2021	
405	cost	Understanding the barriers and enablers to using outdoor spaces in nursing homes: A systematic review	2020	
406	cost	Understanding the long-term impact of flooding on the wellbeing of residents: A mixed methods study	2022	
407	wrong person group	Understanding the Perspectives of Latino Adults Who Smoke on Physical Activity: A Qualitative Study	2023	
408	cost	Understanding weather and hospital admissions patterns to inform climate change adaptation strategies in the healthcare sector in uganda	2018	
409	cost	Urban greenness and mortality in Canada's largest cities: a national cohort study	2017	
410	cost	Use of mobile applications and health technologies among dementia caregivers with chronic conditions: A cross-sectional study.	2023	
411	wrong person group	Using concept mapping to prioritize barriers to diabetes care and self-management for those who experience homelessness	2021	
413	cost	Using technology to deliver healthcare education to rural patients.	2011	
414	care	Using the revenues from carbon pricing - Insights into the acceptance and perceptions of particularly burdened groups	2023	

416	wrong person group	Voices and perspectives of rural Hispanic households on food insecurity in west Texas: a qualitative study	2019	
417	wrong person group	Vulnerability assessment based on household views from the Dammar Char in Southeastern Bangladesh	2022	
418	cost	Vulnerability to the mortality effects of warm temperature in the districts of England and Wales	2014	
419	wrong person group	Vulnerable, Resilient, or Both? A Qualitative Study of Adaptation Resources and Behaviors to Heat Waves and Health Outcomes of Low-Income Residents of Urban Heat Islands.	2022	
420	climate change	WAP explained	2004	
421	wrong person group	Weathering the rural reality: Delivery of the Nurse-Family Partnership home visitation program in rural British Columbia, Canada	2019	
422	wrong person group	What can we learn from Australian general practices taking steps to be more environmentally sustainable? A qualitative study	2023	
423	duplicate	What do we know about the healthcare costs of extreme heat exposure? A comprehensive literature review.	2019	Systematic review and relevant results are from Toloo are already included
424	wrong person group	What influences physical activity in people with heart failure?: a qualitative study.	2011	
425	cost	Who emits most? Associations between socio-economic factors and UK households' home energy, transport, indirect and total CO2 emissions	2013	
426	wrong person group	Willingness to help climate migrants: A survey experiment in the Korail slum of Dhaka, Bangladesh	2021	
427	care	Willingness to pay for policies to reduce future deaths from climate change: evidence from a British survey	2019	
428	care	Willingness to pay for protection from storm surge damages under climate change in Halifax Regional Municipality	2019	
