# Supplementary material for: Climate change, trending outcomes for the care of older people, and financial expenditure: a systematic review and narrative synthesis
Source: BMC Public Health. 2026 Apr 23;26:1355. doi: 10.1186/s12889-026-27435-9 (PMC13107738; doi:10.1186/s12889-026-27435-9)
Supplement: Supplementary file 4 — Supplementary Material 4. [file 12889_2026_27435_MOESM4_ESM.pdf]

Presentation of the selected studies

Citation	Author, year	Title	Location of Study	Study design	Sample size(s)	Exposure(s)	Related expenditure	Main conclusions	MMAT/ CASP checklist
58	Astill & Miller (2018)	The trauma of the cyclone has changed us forever': Self-reliance, vulnerability and resilience among older Australians in cyclone-prone areas	Australia<br><br>Communities between Babinda and Tully Heads North Queensland	interview survey<br>focus groups	n= 16<br>65-79<br><br>n= 20<br>80-94	cyclone	cyclone preparation (extra food, water, clothes), insurance, cyclone recovery (repairs), rebuilding public infrastructure	- having limited funds affect both the ability to prepare and post-cyclone resilience<br>- older people are reliant both on support from community and on government due to limited physical abilities	qualitative
55	Belza et al. (2004)	Older adult perspectives on physical activity and exercise: voices from multiple cultures	USA<br><br>Seattle, Washington area	focus group	n=71	inclement weather	physical activity, transportation	- low or no cost physical activity classes should be offered to increase participation<br>- adverse weather is a barrier to physical activity<br>- specific measures are needed to include ethnically diverse groups	qualitative
47	Chau et al. (2008)	Valuing the health benefits of improving indoor air quality in residences	Hong Kong<br><br>"most polluted neighborhood"	intervention study	population of Hong Kong (unspecified)	seasonality	hospital admission, public services (medical subsidy)	- using an air cleaner with closed windows in winter resulted in the most impact<br>- behavior change attributed to positive outcomes	economic
56	Clemens et al. (2013)	Summer of sorrow: measuring exposure to and impacts of trauma after Queensland's natural disasters of 2010-2011	Australia<br><br>Queensland	cross-sectional survey, quantitative	n= 12,564 survey respondents, all ages	floods, cyclones	property damage (to own home or income-producing property)	- retirees less likely to report being affected by damage due to less home ownership<br>- seniors least likely to worry how they will manage<br>- seniors least terrified<br>- seniors least likely to think they will be injured or die<br>-less likely to report income loss<br>- working- age reported to be more affected	cohort
59	Cooper et al. (2020)	Multimodal physical activity participation rates in middle-aged and older adults	Ireland<br><br>County Laois	survey (quantitative), cross-sectional	n=353	inclement weather	physical activity	- adverse weather a barrier to physical activity<br>- costs not a barrier<br>- outside activities top choice of physical activity for both middle-aged and older adults	cohort
48	Cotter et al. (2012)	Coping with the cold - Exploring relationships between cold housing, health and social wellbeing in a sample of older people in Ireland	Ireland	survey (quantitative) with notes field (qualitative)	n=722	cold weather, extreme cold	fuel (for heating)	-associations observed between living in cold home and higher levels of chronic illness, falls, loneliness, and few social activities	MMT
51	Daum & Dobrof (1983)	Seasonal vulnerability of the old and cold: The role of the senior citizen center	USA<br><br>New York State	questionnaire	n= 162<br><br>directors of senior citizen centers surveyed	seasonality	fuel (for heating senior centers), personnel (extra demand during winter)	- transportation is a major issue for seniors in winter, compounded by illness and getting to appointments.<br>- more resources are needed in winter to meet seasonal increase in demand for services	MMAT
64	Duncombe et al. (2001)	Retire to where? A discrete choice model of residential location	USA<br><br>(continental)	cross-sectional observational study	n=3069 US counties from 48 states	warm weather, cold weather, humidity	housing/ living, taxes, public services (medical, fire, police, recreation)	- high value placed on weather when deciding where to retire<br>- costs for housing and low taxes are valued	cohort

Citation	Author, year	Title	Location of Study	Study design	Sample size(s)	Exposure(s)	Related expenditure	Main conclusions	MMAT/ CASP checklist
50	Hansen et al. (2022)	The Thermal Environment of Housing and Its Implications for the Health of Older People in South Australia: A Mixed-Methods Study	Australia<br><br>3 regions in South Australia	mixed- method<br><br>survey, focus groups, house monitoring	n= 303<br><br>n= 250, survey<br>n= 49, focus group<br>n=71, home study monitoring	extreme heat, extreme cold, humidity	energy (electricity, gas for heating/cooling)	- self-rated health correlated to optimal thermal comfort. Worse health in extreme cold and extreme heat.<br>- higher relative humidity associated with lower temperatures but perception of better health/wellbeing<br>- elevated temperatures associated with higher health and wellbeing<br>- costs of heating/ cooling barrier for older adults	MMT
70	Huang et al. (2023)	Heatwave and urinary hospital admissions in China: Disease burden and associated economic loss, 2014 to 2019	China	epidemiological study: time-stratified case-crossover method with distributed lag nonlinear model	n=23<br>study sites in 4 climate zones<br><br>n= 96092 admission cases	heatwave	hospital admission	- higher risk of hospitalizations from heatwaves for people aged 15-64<br>- 1463 patients every year hospitalized for urinary diseases attributable to heatwaves from May-Sept.	case control
65	James (2017)	Impacts of leaky homes and leaky building stigma on older homeowners	New Zealand	mixed method<br><br>semi-structured interviews<br>quantitative data analysis	n=12, in-depth interviews<br><br>triangulated by housing price data and data on compensation	rainfall, humidity	house repair, private and public burden (dispute and compensation)	- leaky homes lead to a reduction in the standard of living for older people<br>- leads to reduction in assets of home, which has implications for residential care subsidies for long-term residential care	MMT
58	Kammerbauer & Wamsler (2017)	Social inequality and marginalization in post-disaster recovery: Challenging the consensus?	Germany<br><br>Deggendorf; Fischerdorf and Natternberg districts	interview, documentation review, walk-through analysis, survey, observation, geographical analysis	n=53*<br>households participating in survey<br><br>n=8<br>interview with key informants<br><br>* not stratified for age	floods	house repair, temporary housing	- seniors benefited from government support after flood<br>- seniors and migrants highly affected (either supported or further marginalized) depending on type of assistance offered<br>- seniors reliant on community volunteers for filling out application forms	MMT
42	Lai et al. (2023)	Perception of extreme hot weather and the corresponding adaptations among older adults and service providers-A qualitative study in Hong Kong	Hong Kong<br><br>Tai Po district	focus group, semi-structured interview, thematic analysis	n=46, older adults<br><br>n=18, staff members<br><br>n=2, district councilors	extreme heat	electricity	- costs for electricity along with low income during retirement a concern<br>- older adults do not perceive that they are at risk<br>- indoor facilities to support older people during hot weather are needed	qualitative
43	Lane et al. (2014)	Extreme heat awareness and protective behaviors in New York City	USA<br><br>New York City	quantitative and qualitative methods<br><br>phone survey, focus groups	Survey<br>50-64: n=169 (22%)<br><br>65+: n=186 (16%)	extreme heat, heat waves	electricity, AC unit	- gaps in personal risk perception<br>- 65+ most vulnerable to heat, but were least likely to recognize heat warnings	MMAT

Citation	Author, year	Title	Location of Study	Study design	Sample size(s)	Exposure(s)	Related expenditure	Main conclusions	MMAT/ CASP checklist
72	Liu et. al (2019)	Degrees and dollars – Health costs associated with suboptimal ambient temperature exposure	USA<br><br>Minneapolis/St. Paul Twin Cities Metropolitan Area	epidemiological study<br><br>lag non-linear model	senior age group: mortality, n=225,614 morbidity, n=720,096 ED visit + hospital admission, n=587,343	extreme heat, extreme cold	medical care (emergency department visits and hospital admission)	-suboptimal temperature associated with mortality burden among seniors and high health-related economic costs in urban settings<br>-suboptimal low temperature contributed to more health-related economic costs than suboptimal high temperature<br>-seniors have large numbers of temperature-related mortality and ED visits<br>-majority of economic costs attributed to cold (75%)	case control
69	Noe et al. (2012)	Exposure to natural cold and heat: Hypothermia and hyperthermia Medicare claims, United States, 2004-2005	USA	epidemiological study<br><br>compared 3 data sets for analysis	n=18,768*<br><br>*includes 7 million people younger than 65 who were disabled (16%)	extreme heat, extreme cold	burden on Medicare system (public health service for 65+ in US)<br><br>includes emergency department visits, intensive care unit, skilled nursing facility, and outpatient visits	- preventable conditions among older adults' place a burden on the Medicare system<br>-targeted preparedness activities for extreme weather events are needed<br>-85+ age group is most susceptible and most prone to in-patient medical care	case control
53	Nunes (2018)	The contribution of assets to adaptation to extreme temperatures among older adults	Portugal<br><br>Lisbon	semi-structured interview,<br>data analysis	n= 52, heat-related interviews<br><br>n= 46 cold-related interviews	extreme heat, extreme cold	energy, heating/cooling devices, food, medication	- income is associated with the ability to adapt to both hot/cold temperatures<br>- older adults will forgo other spending or live frugally to heat/cool homes	MMT
40	O'Sullivan et al. (2011)	Making the connection: The relationship between fuel poverty, electricity disconnection, and prepayment metering	New Zealand<br><br>Auckland, Wellington, Christchurch, Dunedin	price comparison analysis*<br><br>semi-structured interview<br><br>*results not stratified for age	n=4<br>interviews	cold weather	fuel, electricity	- older people worry about energy costs and being dependent on them for medical equipment and heating	MMT
71	Rolden et al. (2015)	Seasonal Variation in Mortality, Medical Care Expenditure and Institutionalization in Older People: Evidence from a Dutch Cohort of Older Health Insurance Clients	Netherlands	observational longitudinal study, retrospective<br><br>(seasonal and trend decomposition using Loess method, retrospective)	n = 61,495	seasonality	medical care, institutionalization	- seasonal weather changes impact mortality and medical care expenditure in older people<br>- winter is the most vulnerable time for seniors<br>- costs are highest in summer<br>- institutionalization peaks in winter	cohort
52	Rose et al. (1989)	Comfort and fuel use in 14 South-London pensioners' flats during winter	UK<br><br>South London	survey (quantitative), and temperature measurement	n=14<br><br>single estate in South London	cold weather	fuel for heating	-positive correlation between income and fuel expenditure for heating<br>- for living rooms, reduction in the rate of heat loss (improving insulation or heating system) would result in fuel savings<br>-lower income households were limited in the additional rooms they heated	cohort

Citation	Author, year	Title	Location of Study	Study design	Sample size(s)	Exposure(s)	Related expenditure	Main conclusions	MMAT/ CASP checklist
60	Sanders et al. (2018)	Using formative research with older adults to inform a community physical activity programme: Get healthy, get active	UK<br><br>Northwest England	focus groups, thematic analysis	n= 34	seasonality	transportation, physical activity programs	- costs and transportation are barriers to participating in physical activities	qualitative
67	Schmeltz et al. (2016)	Economic burden of hospitalizations for heat-related illnesses in the United States, 2001–2010	USA	observational retrospective study descriptive statistics and cost reports	all Hospitalizations/heat related illness hospitalizations, n= 181,094,795/ n= 73,180 Using the Nationwide Inpatient Sample (NIS), 8 million hospitalizations per year	extreme heat, heat waves	hospitalizations (admissions)	-adults aged 40–64 years old have a large percentage of hospitalizations for heat-related illnesses during the observed period<br><br>-older adults aged 65+ years old had even higher costs for hospitalizations due to heat-related illnesses	cohort
63	Schmidt et al. (2016)	Exploring beliefs around physical activity among older adults in rural Canada.	Canada<br><br>Two rural communities (one town and one neighboring community) in Saskatchewan	qualitative semi-structured interviews, content analysis	n=10	inclement weather, ice, slush, wind	physical activity facilities	- winter is associated with less physical activity due to poor weather conditions (ice) and a fear of falling<br><br>- older adults are concerned about spending money on indoor physical activity	qualitative
56	Seebauer & Winkler (2020)	Should I stay or should I go? Factors in household decisions for or against relocation from a flood risk area	Austria<br><br>Eferding Basin	qualitative<br><br>semi-structured interviews, longitudinal	3 interview rounds conducted*<br>n= 79 unique households from 3 rounds of interviews:<br><br>n=78<br>n=44<br>n=27<br><br>*not stratified for age	floods	relocation, repair	- older people lack the physical stamina for relocation and building new house<br>- economic considerations, like cost of new house limit relocation<br>- older people have emotional attachment to their homes	qualitative
45	Soebarto et al. (2019)	Living environment, heating-cooling behaviours and well-being: Survey of older South Australians	Australia<br><br>3 climate zones (semi-arid, warm, temperate) in South Australia	observational, cross-sectional study (telephone survey (quantitative))	n=250	hot weather, cold weather	air conditioning, heating	- people concerned about costs of heating and cooling<br>-50% of the 70% who are concerned about costs have a low-income<br>- existing buildings can be improved to reduce heating/cooling use and to improve well-being of older people<br>- a lack of insulation (older housing was better insulated) and shading related to use of heater/ AC<br>- well-designed buildings more efficiently heat and cool	cohort
61	Solomon et al. (2018)	Qualitative longitudinal study of episodic disability experiences of older women living with HIV in Ontario, Canada	Canada<br><br>Southern Ontario	qualitative longitudinal study<br><br>semi-structured	n=10	inclement weather, cold weather	living, affordable outdoor exercise activities	- weather conditions pose as a risk factor of social isolation<br>-cold weather increased pain from arthritis<br><br>- costs of activities in winter were a concern	qualitative

Citation	Author, year	Title	Location of Study	Study design	Sample size(s)	Exposure(s)	Related expenditure	Main conclusions	MMAT/ CASP checklist
				interviews over 20 months					
41	Tang & Zolnikov (2021)	Examining opportunities, challenges and quality of life in international retirement migration	international- on 4 continents	systematic literature review	n=22<br><br>studies reviewed	pleasant weather	living, medical care	- push factors for retiring abroad are costs of care and living, good weather, and transportation	systematic review
47	Tod et al. (2012)	Understanding factors influencing vulnerable older people keeping warm and well in winter: A qualitative study using social marketing techniques	UK<br><br>Rotherham, South Yorkshire	semi-structured interviews, focus group	n=50<br><br>n=24<br>group interviews	cold weather, extreme cold	fuel	- older people are at risk of a cold home<br>- personal values can influence decisions which negatively impact health	qualitative
73	Toloo et al. (2015)	Projecting excess emergency department visits and associated costs in Brisbane, Australia, under population growth and climate change scenarios	Australia<br><br>greater Brisbane area	epidemiological study	n= 12 public hospitals<br>n=600,000 patients*<br><br>*not stratified for age	extreme heat, heat waves	emergency department visits	- number of heat-related excess emergency department visits are expected to increase for older people	case control
44	Valente et. al (2022)	Energy poverty, housing and health: the lived experience of older low-income Australians	Australia<br><br>Sydney, Melbourne	qualitative<br><br>semi-structured interviews, thematic analysis	n=23<br><br>people solely or primarily reliant on government Age Pension for their income	extreme heat, extreme cold	energy prices (electricity, gas) to maintain thermal comfort, AC, rent	- energy poverty is a central issue impacting quality of life for low-income, older Australians	qualitative
49	Wagner et al. (1987)	The Kansas City warm room project: Economics, energy savings and health and comfort impacts	USA<br><br>Kansas City, Missouri	workshop, interview, on-site audit/economic analysis	n= 9<br><br>n= 5, received retrofit warm room<br><br>14 occupants total<br>n= 4 control	cold weather	materials and installation of warm rooms, gas, electricity	- weatherization of homes resulted in gas and electricity savings to heat houses	MMT
74	Wang et al. (2021)	Temperatures and health costs of emergency department visits: A multisite time series study in China	China	epidemiological study	n= 24 study sites, each with at least 300,000 residents	extreme heat, extreme cold	emergency department visits	- exposure to heat and cold results in increased health costs for all age groups, however older adults were not the group with highest costs	case control
75	Wu et al. (2021)	Temperature variability and asthma hospitalisation in Brazil, 2000-2015: A nationwide case-crossover study	Brazil	time stratified case-crossover	n= 1,816 cities population coverage 79%	temperature variability (short-term exposure)	hospitalization	- older adults were most vulnerable to temperature variations and asthma hospitalizations than other age groups	case control

Citation	Author, year	Title	Location of Study	Study design	Sample size(s)	Exposure(s)	Related expenditure	Main conclusions	MMAT/ CASP checklist
68	Xu et al. (2022)	Associations of climate variability driven by El Niño-southern oscillation with excess mortality and related medical costs in Chinese elderly	China	longitudinal	n= 30,763<br><br>adults from the Chinese Longitudinal Healthy Longevity Survey (85% of population)	climate variability (driven by El Nino-Southern Oscillation)	medical costs	-climate variability driven by ENSO could be a crucial determinant of healthy aging<br>-extreme ENSO conditions are associated with increased morality risk and medical burden/total medical costs	cohort
66	Yoshida et al. (2022)	Effects of the 2018 Japan Floods on long-term care insurance costs in Japan: retrospective cohort study.	Japan<br><br>Hiroshima, Okayama and Ehime prefectures	retrospective cohort study	long-term care insurance system users<br><br>n=279,578<br><br>flood victims<br>n=3024	floods	long-term care insurance costs	- 2018 Japan Floods led to increases in long-term care insurance costs and short-stay facilities, and a decrease in the use of home-based services<br><br>- total monthly costs increased regardless of location<br>-Changes diminished 6 months after disaster	cohort study
62	You et al. (2021)	Ethnic Differences in Barriers and Enablers to Physical Activity Among Older Adults	Australia	qualitative<br><br>thematic analysis	n=64	adverse weather	physical activity	- targeted programs are needed to increase program participation for older adults<br>- adverse weather conditions and other environmental factors are barriers to exercise	qualitative
