# Supplementary material for: Climate change, trending outcomes for the care of older people, and financial expenditure: a systematic review and narrative synthesis
Source: BMC Public Health. 2026 Apr 23;26:1355. doi: 10.1186/s12889-026-27435-9 (PMC13107738; doi:10.1186/s12889-026-27435-9)
Supplement: Supplementary file 5 — Supplementary Material 5. [file 12889_2026_27435_MOESM5_ESM.pdf]

Quality assessment checklists

Case Control

Adapted from CASP checklist for case studies checklist. Available from:https://casp-uk.net/casp-tools-checklists/

Legend: Y= yes, N= NO, ? = cannot tell, n/a = not applicable
(This checklist is used for epidemiological studies that are not case studies.)

	Are the results valid?						What are the results?		
	Did the study address a clearly focused issue?	Did the authors use an appropriate method to answer their question?	Were the cases recruited/identified in an acceptable way?	Was the exposure accurately measured to minimise bias?	Aside from the experimental intervention, were the groups treated equally?	Have the authors taken account of the potential confounding factors in the design and/or in their analysis?	Do you believe the results?	Comments	
Huang et al. (2023)	Y	Y	Y	Y	Y	Y	Y		epidemiological study
Liu et al. (2019)	Y	Y	Y	Y	Y	Y	Y		epidemiological study
Noe et al. (2012)	Y	Y	Y	Y	Y	Y	Y	secondary diagnosis excluded	epidemiological study
Toloo et al. (2015)	Y	Y	Y	Y	Y	Y	?	Projections on future climate change scenarios can only be estimated	epidemiological study
Wang et al. (2021)	Y	Y	Y	Y	Y	Y	Y	epidemiological study; elderly not more vulnerable to heat, no differences in costs for male/female	epidemiological study
Wu et al. (2021)	Y	Y	Y	Y	Y	Y	Y		epidemiological study

Cohort

Adapted from CASP checklist for cohort studies checklist. Available from:https://casp-uk.net/casp-tools-checklists/

Legend: Y= yes, N= NO, ? = cannot tell, n/a = not applicable
(This checklist is used for cross-sectional, observational, longitudinal, and retrospective observational studies)

	Are the results valid?								What are the results?	
	Did the study address a clearly focused issue?	Was the cohort recruited/identified in an acceptable way?	Was the exposure accurately measured to minimise bias?	Was the outcome accurately measured to minimise bias?	Have the authors identified all important confounding factors?	Have they taken account of the confounding factors in the design and/or analysis?	Was the follow up (of subjects) complete enough?	Was the follow up (of subjects) long enough?	Do you believe the results?	Comments
Clemens et al. (2013)	Y	Y	?	Y	Y	Y	n/a	n/a	Y	Cross-sectional study: no follow-up in study design
Cooper et al. (2020)	Y	Y	N	Y	Y	Y	n/a	n/a	Y	no follow up in study design
Duncombe et al. (2001)	Y	Y	Y	?	Y	Y	n/a	n/a	Y	comparison of cohort census data
Rolden et al. (2015)	Y	?	Y	Y	Y	Y	Y	Y	Y	data retrieved for a period of 42 months; 80+ age group possibly not accurately represented
Rose et al. (1989)	Y	N	Y	Y	N	N	n/a	n/a	Y	small-scale study (n=12), cross sectional design
Schmeltz et al. (2016)	Y	Y	Y	Y	Y	Y	n/a	n/a	Y	
Soebarto et al. (2019)	Y	Y	Y	Y	Y	N	n/a	n/a	Y	Cross-sectional study: no follow-up in study design
Yoshida et al. (2022)	Y	Y	Y	Y	Y	Y	Y	Y	Y	
Xu et al. (2022)	Y	Y	Y	N	Y	Y	Y	Y	Y	missing mortality data includes estimates of medical costs from family

Economic

Adapted from CASP checklist for economic evaluations checklist. Available from:https://casp-uk.net/casp-tools-checklists/

Legend: Y= yes, N= NO, ? = cannot tell, n/a = not applicable

	Is the economic evaluation valid?				What are the results?				
	Was a well-defined question posed?	Was a comprehensive description of the competing alternatives given?	Does the paper provide evidence that the intervention would be effective?	Were the effects of the intervention identified, measured and valued appropriately?	Were all important and relevant resources required, and health outcome costs for each alternative identified, measured in appropriate units and valued credibly?	Were costs and consequences adjusted for different times at which they occurred (discounting)?	Was an incremental analysis of the consequences and cost of alternatives performed?	Was an adequate sensitivity analysis performed?	Comments
Chau et al. (2008)	Y	Y	Y	Y	Y	?	Y	Y	context-specific intervention

Qualitative

Adapted from CASP checklist for qualitative studies checklist. Available from:https://casp-uk.net/casp-tools-checklists/

Legend: Y= yes, N= NO, ? = cannot tell, n/a = not applicable

	Are the results valid?						What are the results?			
	Was there a clear statement of the aims of the research?	Is a qualitative methodology appropriate?	Was the research design appropriate to address the aims of the research?	Was the recruitment strategy appropriate to the aims of the research?	Was the data collected in a way that addressed the research issue?	Has the relationship between researcher and participants been adequately considered?	Have ethical issues been taken into consideration?	Was the data analysis sufficiently rigorous?	Is there a clear statement of findings?	Comments
Astill & Miller (2018)	Y	Y	Y	Y	Y	N	Y	y	Y	
Belza et al. (2004)	Y	Y	Y	Y	Y	N	N	N	Y	
Lai et al. (2023)	Y	Y	Y	Y	Y	N	Y	Y	Y	
Sanders et al. (2018)	Y	Y	Y	Y	Y	N	Y	Y	Y	
Schmidt et al. (2016)	Y	Y	Y	Y	Y	N	Y	Y	Y	n=10
Seebauer & Winkler (2020)	Y	Y	Y	Y	Y	N	N	Y	N	
Solomon et al. (2018)	Y	Y	Y	?	Y	Y	Y	Y	Y	n=10, 2 major themes identified
Tod et al.(2012)	Y	Y	Y	Y	Y	N	Y	Y	N	
Valente et al. (2022)	Y	Y	Y	Y	Y	Y	Y	N	Y	Recruitment strategy adapted to covid-19 restrictions; n=23;
You et al. (2021)	Y	Y	Y	Y	Y	N	Y	Y	Y	

Systematic

Adapted from CASP checklist for systematic studies checklist. Available from:https://casp-uk.net/casp-tools-checklists/

Legend: Y= yes, N= NO, ? = cannot tell, n/a = not applicable

	**Are the results of the review valid?**					**Will the results help locally?**	
	Did the review address a clearly focused question?	Did the authors look for the right type of papers?	Do you think all the important, relevant studies were included?	Did the review's authors do enough to assess quality of the included studies?	If the results of the review have been combined, was it reasonable to do so?	Were all important outcomes considered?	Comments
Tang & Zolnikov (2021)	Y	Y	?	N	Y	Y	n=22; not stated which databases were searched; quality of studies not assessed

MMT

Adapted from the Mixed Methods Appraisal Tool (MMAT) Version 2018. Available from: http://mixedmethodsappraisaltoolpublic.pbworks.com/w/page/24607821/FrontPage

Legend: Y= yes, N= NO, ? = cannot tell, n/a = not applicable

	MMAT					
	Is there an adequate rationale for using a mixed methods design to address the research question?	Are the different components of the study effectively integrated to answer the research question?	Are the outputs of the integration of qualitative and quantitative components adequately interpreted?	Are divergences and inconsistencies between quantitative and qualitative results adequately addressed? (no divergence is answered with Y)	Do the different components of the study adhere to the quality criteria of each tradition of the methods involved?	Comments
Cotter et al. (2012)	Y	Y	Y	Y	N	Survey (quantitative) with comments field (qualitative); some groups may be overrepresented
Daum & Dobrof (1983)	Y	Y	Y	Y	?	Survey (quantitative) with comments field (qualitative)
Hansen et al. (2022)	Y	Y	Y	Y	Y	Survey and house monitoring (quantitative), focus groups (qualitative)
James (2017)	Y	Y	Y	Y	?	Semi-structured interviews (qualitative) and data analysis (quantitative); n=12
Kammerbauer & Wamsler (2017)	Y	Y	Y	Y	N	Interview (qual.) ,documentation review, walk-through analysis, survey (quant./ qual.), observation, geographical analysis; no information about missing responses or response rate
Lane et al. (2014)	Y	Y	Y	Y	Y	Phone survey (quantitative) and focus groups (qualitative)
Nunes (2018)	Y	Y	Y	Y	Y	Semi-structured interviews (qualitative) and data analysis (quantitative)
O'Sullivan et al. (2011)	Y	?	?	Y	N	Price comparison analysis (qualitative) and semi-structured interview (qualitative, n=4)
Wagner et al. (1987)	Y	Y	Y	Y	N	On-site audit (quantitative) and interview (qualitative)
